# MA-5 attenuates disease-associated transcriptomic aging and pathological microglial states in Gaucher disease

**DOI:** 10.21203/rs.3.rs-10994808/v1

**Published:** 2026-09-14

**Authors:** Yoshiyasu Tongu, Tomoko Kasahara, Kohta Nakamura, Alexander Tyshkovskiy, Yoshitsugu Oikawa, Tetsuro Matsuhashi, Chikahiko Numakura, Risako Fujita, Yoshiteru Kagawa, Zhiqian Yu, Tomoyuki Furuyashiki, Rina Inoue, Daisuke Saigusa, Ryota Kujirai, Yotaro Matsumoto, Eiji Kakazu, Masaru Shimura, Yohei Sugiyama, Shun Watanabe, Yohei Honkura, Kuniyasu Niizuma, Hidenori Endo, Ko Hashimoto, Chiharu Kawabe, Hidetaka Tokuno, Koichi Kikuchi, Chitose Suzuki, Naoyuki Matsumoto, Junken Aoki, Kei Murayama, Hiroaki Tomita, Yuji Owada, Yukio Katori, Tomoyoshi Soga, Yoshihisa Tomioka, Takeya Sato, Takehiro Suzuki, Takafumi Toyohara, Yasushi Okazaki, Paul Anderson, Vadim N. Gladyshev, Takaaki Abe

**Affiliations:** 1. Department of Clinical Biology and Hormonal Regulation, Tohoku University Graduate School of Medicine, Sendai, Japan.; 2. Diagnostics and Therapeutics of Intractable Diseases, Intractable Disease Research Center, Graduate School of Medicine, Juntendo University, Tokyo, Japan.; 3. Division of Genetics, Department of Medicine, Brigham and Women’s Hospital, Harvard Medical School, Boston, MA, USA; 4. Department of Pediatrics, Tohoku University Graduate School of Medicine, Sendai, Japan.; 5. Department of Pediatrics, Yamagata University Faculty of Medicine, Yamagata, Japan; 6. Department of Clinical Genomics, Saitama Medical University; 7. Department of Organ Anatomy, Tohoku University Graduate School of Medicine, Sendai, Japan.; 8. Florey Institute of Neuroscience and Mental Health, University of Melbourne, Parkville, VIC, Australia; 9. Department of Psychiatry, Graduate School of Medicine, Tohoku University, Sendai, Japan.; 10. Department of Pharmacology, Graduate School of Medical and Dental Sciences, Institute of Science Tokyo; 11. Laboratory of Biomedical and Analytical Sciences, Faculty of Pharmaceutical Sciences, Teikyo University, Tokyo, Japan.; 12. Laboratory of Oncology, Pharmacy Practice and Sciences, Tohoku University Graduate School of Pharmaceutical Sciences, Sendai, Japan; 13. Department of Liver Diseases, The Research Center for Hepatitis and Immunology, National Center for Global Health and Medicine, Chiba, Japan.; 14. Department of Metabolism, Chiba Children’s Hospital, Chiba, Japan.; 15. Department of Pediatrics, Juntendo University, Tokyo, Japan.; 16. Department of Nephrology and Hypertension, Tohoku University, Sendai, Japan.; 17. Department of Otolaryngeal Surgery, Tohoku University Graduate School of Medicine, Sendai, Japan; 18. Department of Neurosurgical Engineering, Graduate School of Biomedical Engineering, Tohoku University; 19. Department of Translational Neuroscience, Tohoku University Graduate School of Medicine; 20. Department of Neurosurgery, Tohoku University Graduate School of Medicine, Sendai, Japan; 21. Department of Orthopedic Surgery, Tohoku University Graduate School of Medicine, Sendai, Japan; 22. Department of Health Chemistry, Graduate School of Pharmaceutical Sciences, The University of Tokyo, Tokyo, Japan; 23. Human Biology-Microbiome-Quantum Research Center, Keio University, Tsuruoka, Japan; 24. Division of Rheumatology, Inflammation, and Immunity at Brigham and Women’s Hospital, Harvard Medical School, Boston, MA, USA; 25. Broad Institute, Cambridge, MA, USA; 26. Division of Medical Science, Tohoku University Graduate School of Biomedical Engineering, Tohoku University Graduate School of Medicine, Sendai, Japan

**Keywords:** Gaucher disease, microglia, inflammation, cGAS-STING pathway, NLRP3 inflammasome, mitochondria, Aging clock (tAge), galectin-3

## Abstract

Chronic inflammation and mitochondrial dysfunction are hallmarks of neurodegeneration and aging, yet how mitochondrial damage contributes to inflammatory and aging-like cellular programs remains poorly understood. Gaucher disease (GD) is a lysosomal storage disorder caused by GBA1 mutations, which also represent a major genetic risk factor for Parkinson’s disease. In a GD mouse model, we identified marked mitochondrial cristae disorganization accompanied by mitochondrial DNA release, activation of the cGAS–STING and NLRP3 inflammasome pathways, and subsequent inflammatory microglial activation. Circulating galectin-3 was also elevated in both model mice and patients with GD, supporting its potential relevance as a systemic marker of disease-associated inflammation. Mitochonic acid-5 (MA-5) is a mitochondria-targeting compound that interacts with mitofilin/MIC60 in the mitochondrial inner membrane and enhances ATP production. In a GD mouse model and patient-derived iPSC microglia, MA-5 increased ATP levels, preserved mitochondrial cristae integrity, limited mtDNA release, and suppressed cGAS–STING and NLRP3 inflammasome signaling, thereby attenuating microglial innate immune activation and galectin-3 expression. MA-5 also prolonged survival and reversed accelerated transcriptomic aging across multiple brain cell types, with particularly prominent effects in microglia. In the liver, GD was similarly associated with inflammatory, stress-related, and aging-associated transcriptional changes, whereas MA-5 improved liver function, restored mitochondrial morphology, suppressed inflammatory and stress responses, restored metabolic programs, and reduced transcriptomic age. Overall, MA-5 suppresses neuroinflammation and reverses accelerated transcriptomic aging, supporting its potential as a therapeutic strategy for GD and other lysosomal storage disorders, as well as for diseases characterized by mitochondrial dysfunction, chronic inflammation, and pathological accumulation.

## Introduction

Chronic inflammation is a central driver of aging and age-associated diseases^1,2^. In particular, persistent low-grade inflammatory signaling, often referred to as “inflammaging,” has been implicated in the pathogenesis of multiple neurodegenerative disorders, including Parkinson’s disease (PD) and Alzheimer’s disease (AD)^3,4^. Recently, mitochondrial dysfunction has emerged as a major source of sterile inflammatory signaling^5,6^. Under normal conditions, mitochondrial DNA (mtDNA) is confined within the mitochondrial matrix and sequestered from the cytosol by the mitochondrial membranes^7^. In contrast, pathological mitochondrial stress can compromise mitochondrial integrity and induce the release of mtDNA into the cytosol, where mtDNA functions as a damage-associated molecular pattern and activates innate immune pathways such as the cyclic GMP–AMP synthase–stimulator of interferon genes (cGAS–STING) pathway and the NLRP3 inflammasome^6,8^. Thus, preserving mitochondrial integrity and suppressing mtDNA-driven innate immune activation may provide a therapeutic strategy for neurodegenerative diseases.

Gaucher disease (GD), the most prevalent lysosomal storage disorder, is caused by mutations in the glucocerebrosidase gene (GBA1), leading to the accumulation of glucosylceramide (GlcCer) and glucosylsphingosine (Glc-Sph)^9,10^. While type 1 GD primarily affects visceral organs, neuronopathic GD (types 2 and 3) is characterized by severe neurodegeneration and microglial activation^11,12^. In addition, GBA1 mutations are found in approximately 5–15% of patients with PD, making GBA1 one of the strongest genetic risk factors for PD^13,14^.

Although enzyme replacement therapy is effective for the systemic manifestations of GD, currently available enzymes do not cross the blood–brain barrier and therefore have limited efficacy against the neurological manifestations of neuronopathic GD. Recent studies further demonstrate that glucocerebrosidase deficiency promotes mitochondrial dysfunction and mtDNA release in microglia^15^. These findings raise the possibility that mitochondria-modulating strategies may suppress neuroinflammation and microglial activation in neuronopathic GD, GBA1-associated PD, and other lysosomal storage disorders.

We recently developed mitochonic acid 5 (MA-5), a small-molecule compound that enhances mitochondrial ATP production by facilitating ATP synthase oligomerization^16,17^. MA-5 binds to mitofilin/IMMT (Mic60) in the mitochondrial inner membrane, modulates cristae architecture, and improves mitochondrial morphology and dynamics, thereby promoting efficient ATP synthesis, reducing mitochondrial ROS generation, and preserving mitochondrial structural integrity^18^. Currently, a phase II clinical trial of MA-5 for mitochondrial disease is underway (jRCT2031250505). Here, using a mouse model of GD, we show that MA-5 suppresses neuroinflammation, particularly microglial activation; reverses accelerated transcriptomic aging, as measured by our recently developed transcriptomic aging clock, tAge^19^; improves motor function, muscle strength, hearing, and liver function; and prolongs survival.

## Results

### MA-5 ameliorates microglial inflammation in a mouse model of GD

**Figure 1.**
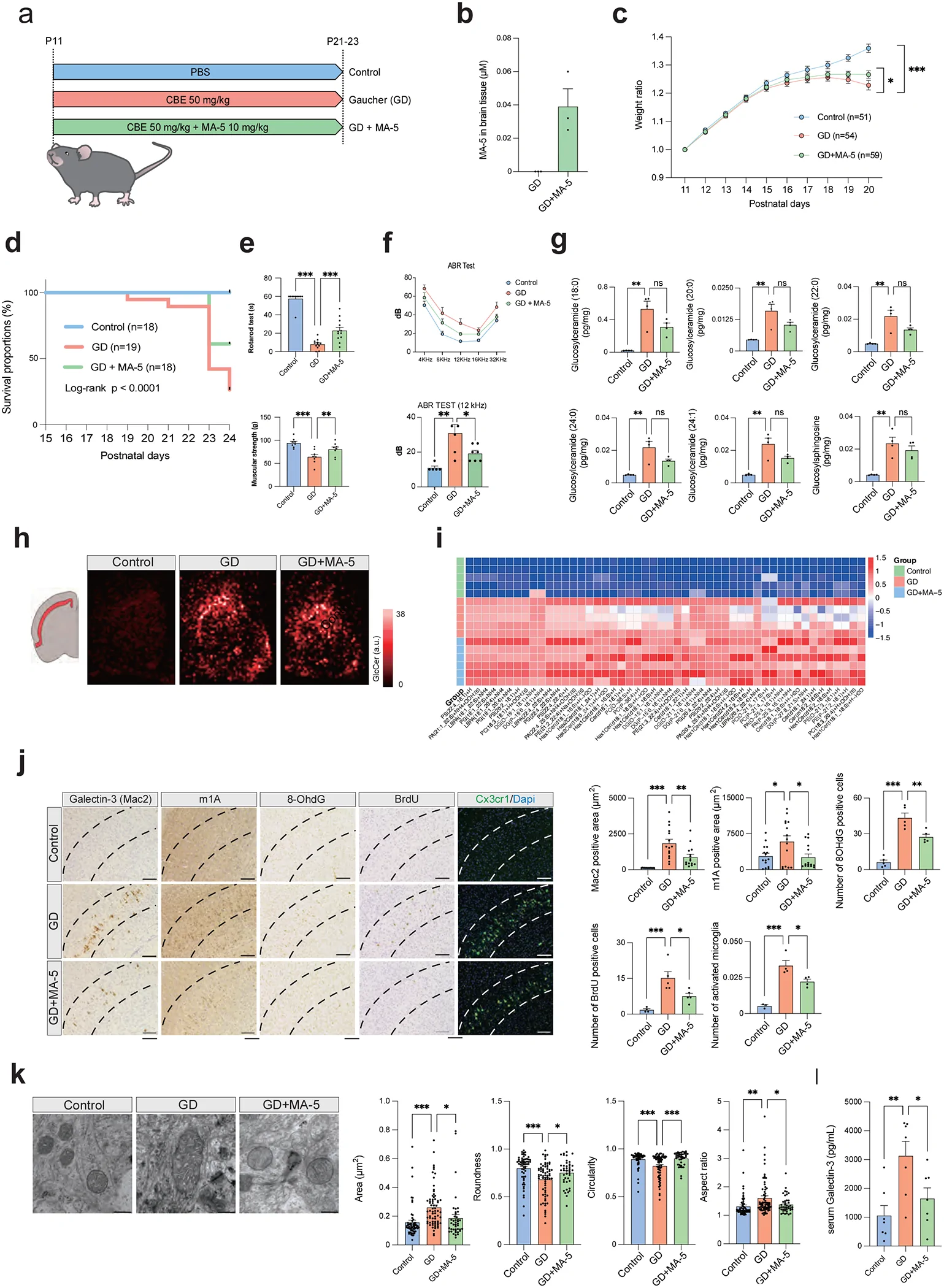
MA-5 ameliorates disease phenotypes and neuroinflammation in GD mice. (a) *In vivo* experiments were performed according to the indicated schedule and dosing regimen, with mice sacrificed around postnatal day 22 (P22), except for survival experiments. (b) The brain concentration of MA-5. Tissue concentration of MA-5 was measured to confirm its ability to cross the BBB (Control, n = 3; GD, n = 3; GD+MA-5, n = 3). (c) Body weight changes from P11 to P20 in Control (n=10), GD (n = 10), and GD+MA-5 (n=10) mice. (d) Kaplan-Meier survival curves for Control (n=18), GD (n=19), and GD+MA-5 (n=18) mice. Statistical significance was determined by log-rank test (*p*<0.0001, GD+MA-5 vs GD). (e) Rotarod test (upper) and muscle strength measurements (lower) in Control, GD, and GD+MA-5 mice (Control, n = 10; GD, n = 12; GD+MA-5, n = 12). Representative images of mice during the rotarod test are shown (**Supplementary movie 2**), (f) ABR test at 4, 8, 12, 16 and 32 kHz of WT and GD mice administrated vehicle (water) or MA-5 water suspension (1mg/kg) for 13 days from postnatal day 28 (P28) to P40 (n=5) (upper). At 12 kHz, GD mice exhibited impaired hearing compared with controls, which was improved by MA-5 treatment (lower). Data were mean ± SE. *p<0.05 vs. vehicle; statistics were calculated by one-way ANOVA Dunnett’s multiple comparison test. (g) Quantification of GlcCer and glucosylsphingosine species in brain tissue (Control, n = 4; GD, n= 4; GD+MA-5, n=4). (h) Imaging mass spectrometry of GlcCer distribution in the cerebral cortex. Scale bars =1 mm. (i) Heatmap of lipidomics data from mouse brains across the three groups (Control, n = 5; GD, n = 5; GD+MA-5, n= 6. (j) Histological analyses of the cerebral cortex. Representative image of galectin-3 (Mac2), 1-methyladenosine (m1A), 8-OHdG, BrdU, and Cx3cr1-GFP staining are shown (left panels), with quantification of the Mac2-positive area, m1A-positive area, the number of 8-OHdG-positive cells, BrdU-positive cells, and activated microglia (right panels) are presented in the corresponding graphs (Control, n = 14; GD, n = 14; GD+MA-5, n = 14 mice per group). Scale bars =100μm. (k) Electron microscopy analysis of mitochondria in the cerebral cortex. Mitochondrial area, roundness, circularity, and aspect ratio were quantified (Control, n=59; GD, n = 55; GD+MA-5, n = 42) and the corresponding quantitative data are shown to the right of the representative images. Scale bars = 200 nm. (l) Serum galectin-3 concentration in Control, GD, and GD+MA-5 mice (Control, n = 7; GD, n = 7; GD+MA-5, n = 7). In the three-group comparison, *p<0.05, **p<0.01, one-way ANOVA Tukey’s test.

To examine the effect of MA-5 on GD, we used a chemically induced GD model generated by inhibiting acid β-glucosidase/glucocerebrosidase (GCase) with conduritol-B-epoxide (CBE), which reproduces the key features of GD, including neuropathology, behavioral abnormalities, and gene expression changes^20^. C57BL/6J mice were injected with CBE (50 mg/kg) on postnatal (P) day 11 (P11), and MA-5 (10 mg/kg) was administered simultaneously (Figure 1a). We first examined whether MA-5 crosses the blood–brain barrier (BBB). Brain concentrations of MA-5 increased following administration, demonstrating efficient BBB penetration and distribution within the brain (Figure 1b). GD mice showed progressive weight loss from P18, which was attenuated by MA-5 treatment (Figure 1c). Consistently, GD mice died between P24 and P36, whereas MA-5 treatment prolonged survival (*p* < 0.0001; Figure 1d). Because GD mice exhibit impaired motor behavior^20^, we evaluated motor function. GD mice showed markedly reduced latency to fall in the rotarod test compared with controls (mean – SEM, 8.0 – 1.5 s vs. 57.7 – 2.3 s). MA-5 improved rotarod performance (23.0 – 3.5 s, *p* < 0.001; Figure 1e, upper panel; **Supplemental Movie 1**). Grip strength was also reduced in GD mice (64.5 – 5.5 g vs 94.4 – 3.6 g in controls) and restored by MA-5 (80.7 – 5.0 g, Figure 1e, lower). These findings indicate that MA-5 rescued motor dysfunction in GD mice. Hearing loss has also been reported in patients with type 2 and type 3 GD^21,22^. Therefore, we assessed auditory function in GD mice using the auditory brainstem response (ABR) test (Figure 1f). At 12 kHz, GD mice exhibited impaired hearing compared with controls, which was improved by MA-5 treatment. These findings indicate that MA-5 ameliorates multiple GD phenotypes and prolongs survival.

Because the improvement in GD phenotypes by MA-5 could potentially be explained by reduced glycolipid accumulation, we next examined whether MA-5 altered glycolipid storage in the brain. To confirm glycolipid accumulation in the GD brain, we measured the Glc-Sph and GlcCer levels. GD mice showed marked accumulation of C18-containing Glc-Sph and multiple GlcCer species, including GlcCer (d18:1/16:0–24:1) in the brain (Figure 1g). MS imaging revealed predominant cortical glycolipid accumulation, which was not reduced by MA-5 treatment (Figure 1h). Lipidomic analysis also revealed distinct lipid profiles between control and GD mice, with only modest changes after MA-5 treatment (Figure 1i). These findings indicate that MA-5 does not reduce glycolipid storage or normalize lipidomic alterations, suggesting that its therapeutic effects are mediated by mechanisms independent of lysosomal substrate clearance.

In the GD mouse brain, GlcCer accumulation induces neuronal dysfunction and microglial activation, triggering neuroinflammation^11^. Microglial activation is reportedly most prominent in cortical layer V^20,23^, where galectin-3 (Mac2)-positive microglia are predominantly observed between days 18 and 25 after CBE treatment^20,24^. Therefore, we examined galectin-3 as a marker of microglial activation and neuroinflammation, 1-methyladenosine (m1A)^25^ as an inflammatory marker, 8-hydroxy-2′-deoxyguanosine (8-OHdG) as an oxidative stress marker, and 5-bromo-2′-deoxyuridine (BrdU) a as a cell proliferation marker (Figure 1j). In the GD mouse brain, galectin-3 and m1A levels were increased in cortical layer V, along with elevated 8-OHdG and BrdU levels, indicating enhanced neuroinflammation, oxidative stress, and cell proliferation in this region. These changes were significantly attenuated by MA-5 treatment. Because CX3CR1, a receptor for fractalkine (CX3CL1), is also upregulated in activated microglia in neurodegenerative and inflammatory disease models^26–28^, we used CBE-treated CX3CR1ĜFP/+ mice to visualize microglial inflammation^29^. CBE administration increased CX3CR1-driven GFP-positive microglial accumulation in cortical layer V, whereas MA-5 markedly reduced this response (Figure 1j).

Mitochondrial structure is closely linked to function and is essential for maintaining cellular health^30,31^. Accordingly, disruption of mitochondrial dynamics can promote inflammation and mtDNA mislocalization^32,33^. In GD cortical neurons, altered mitochondrial morphology and function have also been reported^34^. Therefore, we next examined mitochondrial morphology using electron microscopy^35^. In GD mice, the mitochondrial area and aspect ratio were increased, whereas the roundness and circularity were decreased (Figure 1k). These abnormalities were restored to control levels by MA-5, suggesting that MA-5 improves mitochondrial morphology, thereby ameliorating mitochondrial dysfunction and microglial inflammation in GD.

Because galectin-3 was increased in cortical layer V of GD mice and reduced by MA-5, we next asked whether this inflammatory response was also reflected in the circulation. Although galectin-3 is known to be associated with macrophage activation, inflammation, and fibrosis^36^, whether circulating galectin-3 is elevated in GD has remained unclear. We therefore measured serum galectin-3 levels in GD mice. Serum galectin-3 was significantly increased in GD mice and markedly reduced by MA-5 treatment (Figure 1l), indicating that MA-5 suppresses both local and systemic inflammatory responses. Together, these results suggest that MA-5 ameliorates GD phenotypes by improving mitochondrial integrity and suppressing inflammation despite persistent glycolipid accumulation.

### Single-nucleus transcriptomic atlas of the GD cortex resolves cell-type- and cluster-level disease perturbation

**Figure 2.**
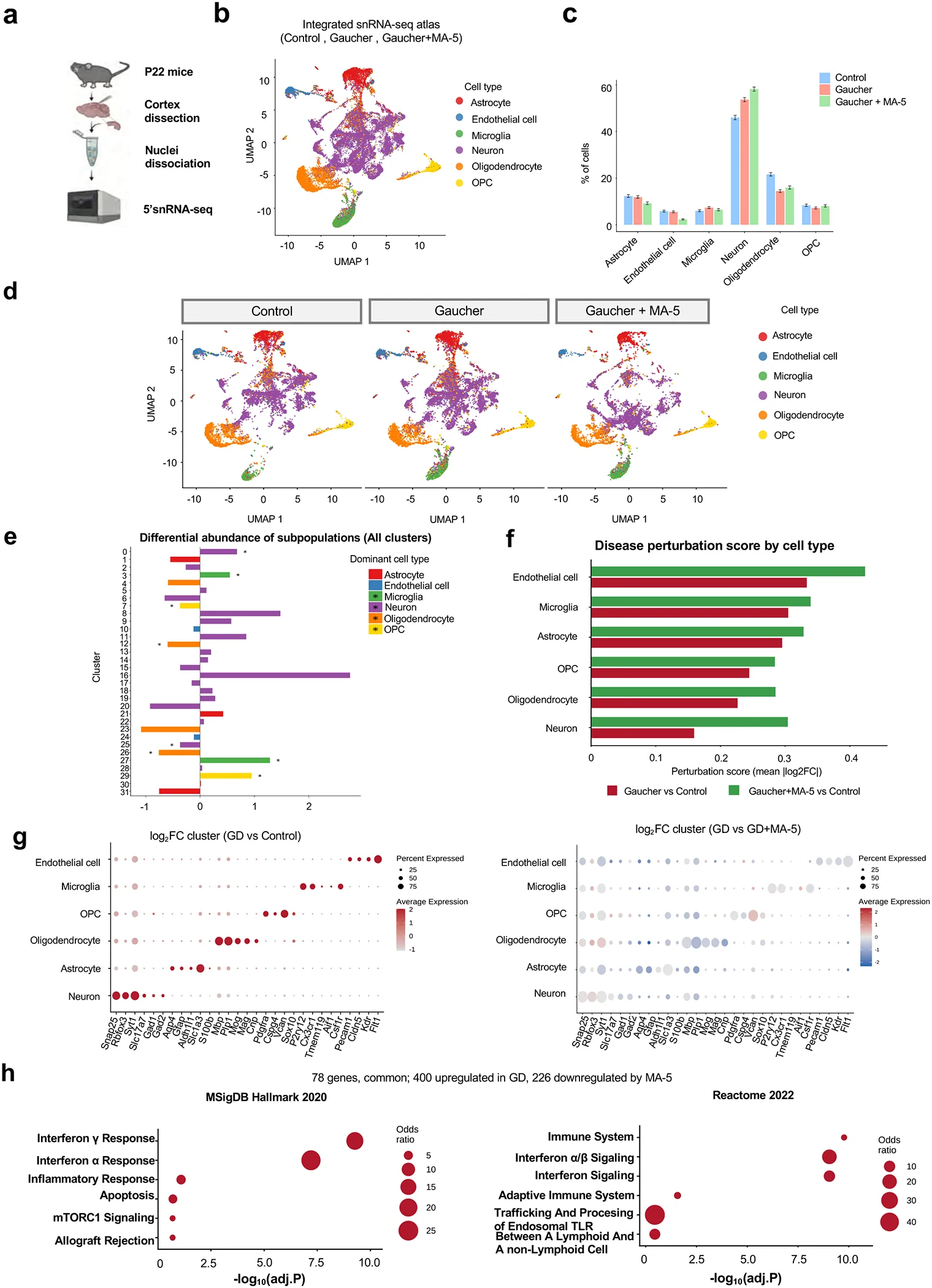
A single-nucleus RNA-sequencing (snRNA-seq) atlas of the GD mouse cortex resolves cell-type- and cluster-level transcriptional perturbation. (a) Schematic of the experimental workflow: cortical dissection, nuclei dissociation, and 5′ snRNA-seq from Control, GD, and GD+MA-5 mice (n = 1 mouse per condition). (b) UMAP embedding of all captured nuclei, integrated across the three conditions, colored by annotated cell type (Astrocyte, Endothelial cell, Microglia, Neuron, Oligodendrocyte, OPC). (c) Relative abundance (% of cells) of each annotated cell type by condition. Error bars denote 95% confidence intervals from bootstrap resampling of cells within each animal (1,000 resamples); estimates describe a single animal per condition and are not inferential across animals. (d) UMAP embedding as in b, split by condition and colored by cell type.(e) Differential abundance of the 32 unsupervised transcriptional clusters, expressed as log2 fold-change in relative cluster size (GD versus Control). Bars are colored by each cluster’s dominant annotated cell type; asterisks denote clusters with a statistically significant shift in relative abundance (see Methods). (f) Disease perturbation score (mean |log2 fold-change| across all detected genes, relative to the condition-matched Control centroid) for each cell type, shown for GD versus Control (red) and GD+MA-5 versus Control (green). Significance was assessed by a within-cell-type label-permutation test (200 permutations); because condition and animal are fully confounded at n = 1 mouse per condition, permutation p-values saturate at the test floor for every cell type and are not used to rank cell types (see Methods). (g) Dot plots of canonical marker genes used for cell-type annotation (left) and their expression changes following MA-5 treatment (right). Dot size indicates the percentage of cells expressing each gene; color indicates scaled average expression (left) or expression change relative to GD (right). (h) Pathway enrichment analysis of the 78 genes upregulated in GD and downregulated by MA-5, showing enrichment of interferon and inflammatory pathways using MSigDB Hallmark 2020 (left) and Reactome 2022 (right). Bubble size indicates the odds ratio.

Because GD pathology in the central nervous system is likely to involve cell-type-specific responses rather than a uniform tissue-wide effect, we first constructed a single-nucleus transcriptomic atlas of the cerebral cortex to identify the cell populations most affected by GD and to characterize their responses to MA-5. We performed 5′ single-nucleus RNA sequencing (snRNA-seq) on cortical tissue from control, CBE-induced GD, and GD+MA-5 mice at postnatal day 22 (Figure 2a). Initially, to determine whether the CBE-induced GD model recapitulates the microglial transcriptional signatures observed in genetic GD models, we compared its transcriptomic profile with those from two independent genetic GD datasets (**Supplementary Figure 1**). Comparison with the scRNA-seq dataset of Boddupalli et al.^37^ (**Supplementary Figure 1a)** and the sorted microglial RNA-seq dataset of Shimizu et al.^38^ (**Supplementary Figure 1b)** revealed strong concordance between CBE-induced and genetic GD microglia (Pearson’s r = 0.742 and 0.741, respectively). Both comparisons showed strong induction of the DAM-associated gene Gpnmb, which exhibited the highest concordance across the datasets. The interferon-related genes Ligp1, Oas1, and Ifi44 were also strongly upregulated in both datasets. Additional interferon-related genes showed strong concordance within each dataset, including Ifi204, Ifi206, Ifi207, Ifi211, and Oas1g and Rai14 in the Boddupalli et al. dataset, and Cxcl10, Gbp6, Irf7, Ifit3b, and Rsad2 in the Shimizu et al. dataset. Together, these findings indicate that DAM activation and interferon-related transcriptional programs are conserved features of microglial activation across CBE-induced and genetic GD models.

Unsupervised clustering identified 32 transcriptionally distinct clusters, which were visualized in a shared Uniform Manifold Approximation and Projection (UMAP) space and annotated into six major cortical cell types—neurons, oligodendrocytes, astrocytes, oligodendrocyte precursor cells (OPCs), microglia, and endothelial cells—based on canonical marker expression (Figure 2b). Cell-type and cluster-level composition across conditions is summarized in **Supplementary Table S1.** We next examined whether cellular composition differed among conditions. Because each condition was represented by a single animal, relative cell-type abundance was evaluated descriptively (Figure 2c). The microglial fraction increased from 5.98% in control to 7.32% in GD and decreased to 6.42% following MA-5 treatment. In contrast, oligodendrocytes showed the largest compositional change, decreasing from 21.6% in control to 14.5% in GD and remaining low after MA-5 treatment (15.9%). Other major cell types showed more modest or variable changes in relative abundance across conditions. Condition-split UMAPs further illustrated differences in cellular distribution across conditions (Figure 2d). To determine whether these differences were concentrated in specific transcriptional substates, we compared the relative abundance of the 32 unsupervised clusters across conditions (Figure 2e). Two microglia-dominant clusters, clusters 3 and 27, showed prominent expansion in GD relative to control (log2 fold-change 0.55 and 1.29, respectively). Several oligodendrocyte-dominant clusters also decreased, whereas an OPC-dominant cluster expanded. These findings suggest that the cellular changes observed in GD were concentrated within specific transcriptional subpopulations of individual cell lineages rather than reflecting uniform shifts across entire cell types. We then quantified the magnitude of transcriptome-wide perturbation within each major cell type using the mean absolute log2 fold-change across detected genes relative to the corresponding control population (Figure 2f, **Supplementary Table S2**). Endothelial cells and microglia showed the largest transcriptome-wide perturbations in GD (scores 0.334 and 0.305, respectively), followed by astrocytes, OPCs, oligodendrocytes, and neurons. Given the single-animal design for each condition, these scores were interpreted descriptively and used to prioritize cell types for downstream analysis. Notably, the overall transcriptomic difference from control was not consistently reduced by MA-5. This does not necessarily indicate a lack of therapeutic response, because this global metric captures both reversal of GD-associated changes and additional transcriptional responses induced by MA-5. Canonical marker expression further confirmed the identity of all six annotated cell populations (Figure 2g, left), including neuronal markers Snap25, Rbfox3 and Syt1; astrocytic markers Aqp4, Gfap and Aldh1l1; oligodendrocyte markers Mbp, Plp1 and Mog; OPC markers Pdgfra and Cspg4; microglial markers P2ry12, Cx3cr1 and Tmem119; and endothelial markers Pecam1, Cldn5 and Kdr.

To determine whether selective reversal of GD-associated transcriptional change was obscured by the transcriptome-wide metric, we next focused specifically on GD-associated genes across major cell types in untreated GD and GD+MA-5 cortex (Figure 2g, right). Notably, within each major cell type, genes that were elevated in GD relative to control were generally reduced by MA-5 in the same cell type, demonstrating a coordinated attenuation of GD-associated transcriptional changes across major cortical cell populations. This coordinated reversal of disease-associated gene expression paralleled the improvement in GD-related pathological phenotypes observed with MA-5 treatment, suggesting that suppression of these transcriptional programs may contribute to its therapeutic effects. To identify disease-associated transcriptional programs selectively reversed by MA-5, we analyzed genes upregulated in GD and downregulated following MA-5 treatment (Figure 2h). Of 400 genes upregulated in GD relative to control and 226 genes downregulated following MA-5 treatment, 78 genes were shared between the two sets. Hallmark analysis identified enrichment of interferon-γ, interferon-α, and inflammatory response pathways, whereas Reactome analysis highlighted interferon and broader immune-system signaling (**Supplementary Table S3**). These findings indicate preferential suppression of GD-associated inflammatory and interferon responses by MA-5. Together with the pronounced transcriptomic perturbation observed in microglia, these findings prompted us to further characterize disease-associated inflammatory and activation-related transcriptional programs in microglia.

### MA-5 selectively reverses specific GD–associated transcriptional programs in microglia

**Figure 3.**
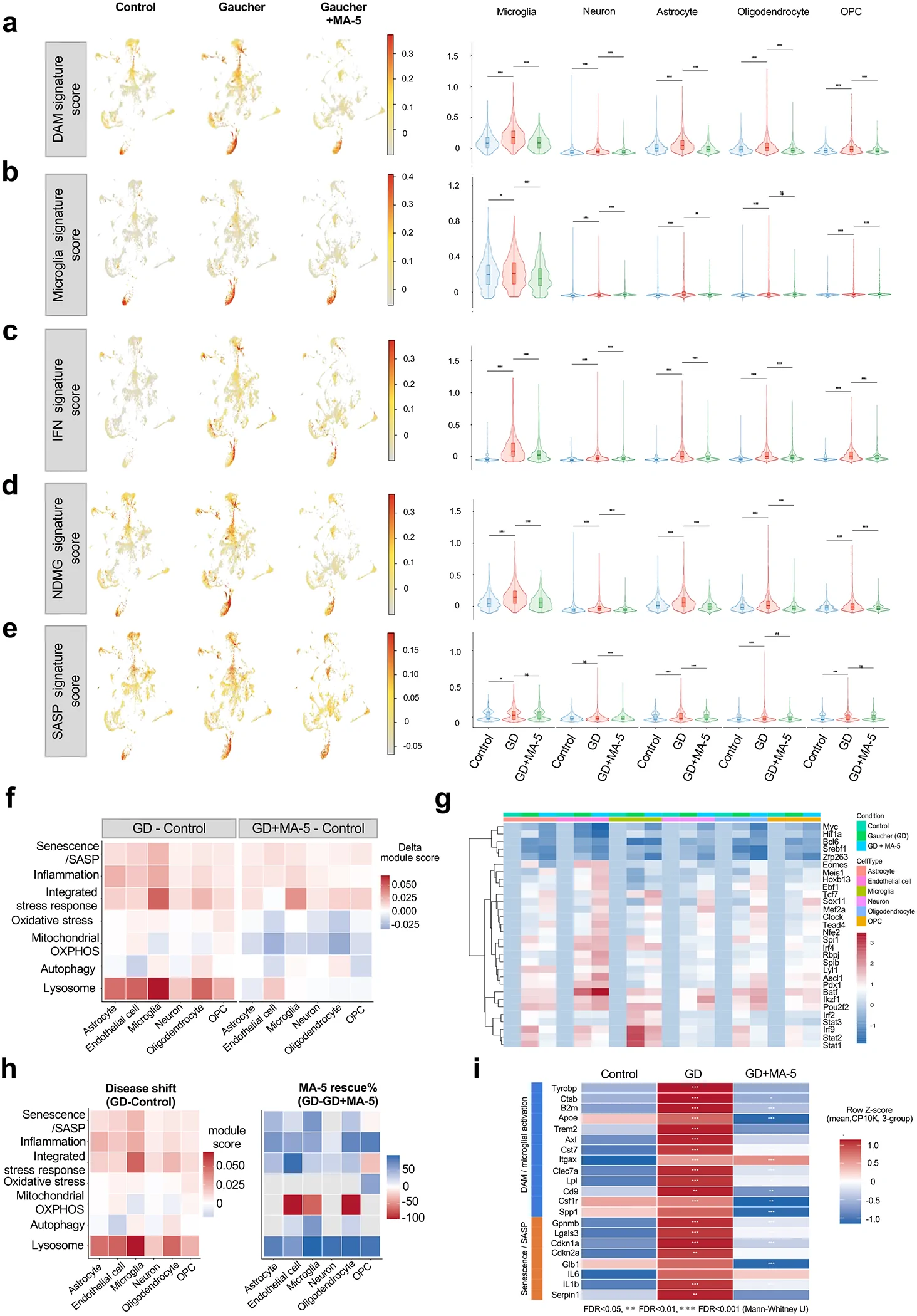
GD-associated pathological transcriptional programs in microglia and their differential reversal by MA-5. a–e, UMAP feature plots, split by condition, of five curated pathological signature module scores (Seurat AddModuleScore): disease-associated microglia (DAM) (a), microglial activation (b), interferon (IFN) response (c), neurodegenerative microglia (NDMG) (d), and senescence-associated secretory phenotype (SASP) (e). Color scale indicates module score; gene lists are provided in **Supplementary Table S4**. (f) Cell-type-specific change in seven curated pathway module scores (Lysosome, Autophagy, Mitochondrial OXPHOS, Oxidative stress, integrated stress response [ISR], Inflammation, Senescence/SASP), expressed as delta module score relative to each cell type’s own Control (GD − Control, left; GD+MA-5 − Control, right). The macrophage population was excluded (see Methods); descriptive (n = 1 mouse/condition). (g) Transcription factor (TF) regulon activity inferred from pseudobulk expression using DoRothEA regulons (confidence A–C) and decoupleR (univariate linear model, ulm), expressed as delta activity from each cell type’s own Control, for all cell-type × condition combinations. Rows are hierarchically clustered by activity pattern; the interferon-responsive ISGF3 complex (Stat1/Stat2/Irf9) clusters together and shows the strongest Gaucher-associated activation, specifically in microglia. (h) MA-5 reversal of GD-induced pathway changes. A, magnitude of the disease-associated shift (GD − Control module score) for each pathway and cell type. B, percentage of that shift reversed by MA-5 (rescue % = 100 × [1 − (GD+MA-5 − Control)/(GD − Control)]); 100% indicates complete rescue, negative values indicate a shift beyond Control in the opposite direction. Values are clipped to [−100, 150]. (i) Heatmap of row-scaled (z-score) mean expression (CP10K) across Control, GD, and GD+MA-5 for a 21-gene panel comprising DAM/microglial-activation markers (Tyrobp, Ctsb, B2m, Apoe, Trem2, Axl, Cst7, Itgax, Clec7a, Lpl, Cd9, Csf1r, Spp1) and senescence/SASP markers (Gpnmb, Lgals3, Cdkn1a, Cdkn2a, Glb1, Il6, Il1b, Serpine1). Asterisks denote FDR-adjusted significance (Mann-Whitney U test on single-cell CP10K values, Benjamini-Hochberg-adjusted) for GD versus Control and GD+MA-5 versus Control (*, FDR<0.05; **, FDR<0.01; ***, FDR<0.001).

Given the prominent transcriptional perturbation and expansion of microglia-dominant clusters observed in Figure 2, we next examined GD-associated pathological transcriptional programs across cortical cell populations and their response to MA-5. We first evaluated five literature-derived gene signatures—DAM, general microglial activation, IFN response, neurodegenerative microglia (NDMG), and senescence-associated secretory phenotype (SASP)—and visualized their scores across the shared UMAP embedding (Figure 3a–e; gene lists in **Supplementary Table S4**). Cell-level comparisons were assessed using Mann–Whitney U tests with Benjamini–Hochberg correction and were interpreted descriptively because each condition was represented by a single animal. All five signatures were increased in GD, with the most pronounced changes observed in microglia. These signatures were attenuated following MA-5 treatment, particularly in microglia, suggesting coordinated suppression of disease-associated inflammatory and senescence-related transcriptional programs. To characterize pathway-level changes associated with GD, we next examined seven curated pathway modules encompassing Lysosome, Autophagy, Mitochondrial oxidative phosphorylation (OXPHOS), Oxidative stress, integrated stress response (ISR), Inflammation, and Senescence/SASP. For each major cell type, changes in module scores were calculated relative to the corresponding control population for GD and GD+MA-5 (Figure 3f, **Supplementary Table S3**). The Lysosome module showed the largest GD-associated increase among the pathways examined, with the strongest change observed in microglia (Δ = +0.069 versus control). The ISR (+0.050) and Inflammation (+0.027) modules were also prominently increased in GD microglia. Following MA-5 treatment, these disease-associated elevations were attenuated across multiple cortical cell types. In contrast, the OXPHOS-associated transcriptional module showed a distinct response, with no apparent shift toward the control state following MA-5 treatment in several cell types. Thus, MA-5 was associated with selective attenuation of specific GD-associated transcriptional programs rather than uniform normalization across pathways.

To identify potential upstream regulators of the GD-associated and MA-5-responsive transcriptional programs, we inferred transcription factor (TF) activity from pseudobulk expression profiles using curated DoRothEA regulons (confidence levels A–C) and the decoupleR univariate linear model (ulm) framework (Figure 3g). TF activities were expressed relative to the corresponding control population for each cell type. Unsupervised hierarchical clustering revealed prominent activation of Stat1, Stat2, and Irf9, which form the interferon-stimulated gene factor 3 (ISGF3) complex. In GD microglia, these factors showed the strongest activation (Δ ≈ +2.8), together with increased Nfkb1 activity (Δ = +1.36). MA-5 partially reduced these activities, implicating ISGF3 and NF-κB as major candidate regulators associated with the interferon and inflammatory state of GD microglia.

To quantify pathway-specific responses to MA-5, we calculated a rescue score representing the proportion of the GD-associated module-score change reversed toward the control level (Figure 3h). A score of 100% indicates complete restoration, 0% no restoration, and negative values a further shift away from control. The Lysosome module showed the strongest restoration, with a rescue score of 99.1% in microglia and similarly high values in oligodendrocytes (94.2%), neurons (90.8%), OPCs (89.0%), and astrocytes (81.7%), with a more moderate response in endothelial cells (65.4%). These results indicate that restoration of the lysosome-associated transcriptional program was broadly shared across major cortical cell types. In contrast, the OXPHOS-associated transcriptional module was not restored toward the control state and instead shifted further below the control baseline following MA-5 treatment in several cell types, resulting in negative rescue scores. This finding reflects a lack of transcriptional restoration of the OXPHOS-associated gene-expression program and does not necessarily indicate impaired mitochondrial respiratory function. In microglia, Inflammation, Autophagy, and Senescence/SASP showed intermediate rescue following MA-5 treatment, with scores of 55.6%, 64.7%, and 65.9%, respectively. These analyses demonstrate that MA-5 selectively reverses GD-associated pathological programs, with particularly strong restoration of lysosomal transcriptional changes and substantial attenuation of autophagy-, inflammation-, and senescence-associated programs.

Finally, to identify individual genes characterizing the pathological microglial response and its modulation by MA-5, we examined a 21-gene panel comprising established DAM/microglial-activation markers (Tyrobp, Ctsb, B2m, Apoe, Trem2, Axl, Cst7, Itgax, Clec7a, Lpl, Cd9, Csf1r, and Spp1) and senescence/SASP-associated genes (Gpnmb, Lgals3, Cdkn1a, Cdkn2a, Glb1, Il6, Il1b, and Serpine1) (Figure 3i). Gpnmb increased approximately 27-fold in GD relative to control and was reduced by approximately 64% following MA-5 treatment, although it remained elevated relative to control. Lgals3, encoding galectin-3, increased approximately 13-fold in GD and was reduced by approximately 56% following MA-5 treatment. Cdkn1a, encoding the cyclin-dependent kinase inhibitor p21, increased approximately 2.3-fold in GD and was reduced by approximately 78% following MA-5 treatment, representing the largest proportional reduction among these three genes. Other senescence/SASP-associated genes showed more heterogeneous responses. Cdkn2a, Il1b, and Serpine1 showed GD-associated induction with partial attenuation following MA-5 treatment, whereas Il6 showed no clear directional change. Glb1 was not substantially altered in GD but decreased below the control level following MA-5 treatment. Together, these findings identify Gpnmb, Lgals3, and Cdkn1a as prominent MA-5-responsive features of the GD-associated microglial state. However, these analyses did not establish whether these GD-associated microglial transcriptional programs were concentrated within a discrete pathological subpopulation or reflected a continuous transition from homeostatic to disease-associated states. We therefore next performed microglia-focused subclustering and trajectory analysis to define the organization of these pathological states and determine how MA-5 altered their distribution.

### MA-5 reduces the GD-associated shift toward a DAM/IFN-enriched microglial state

**Figure 4.**
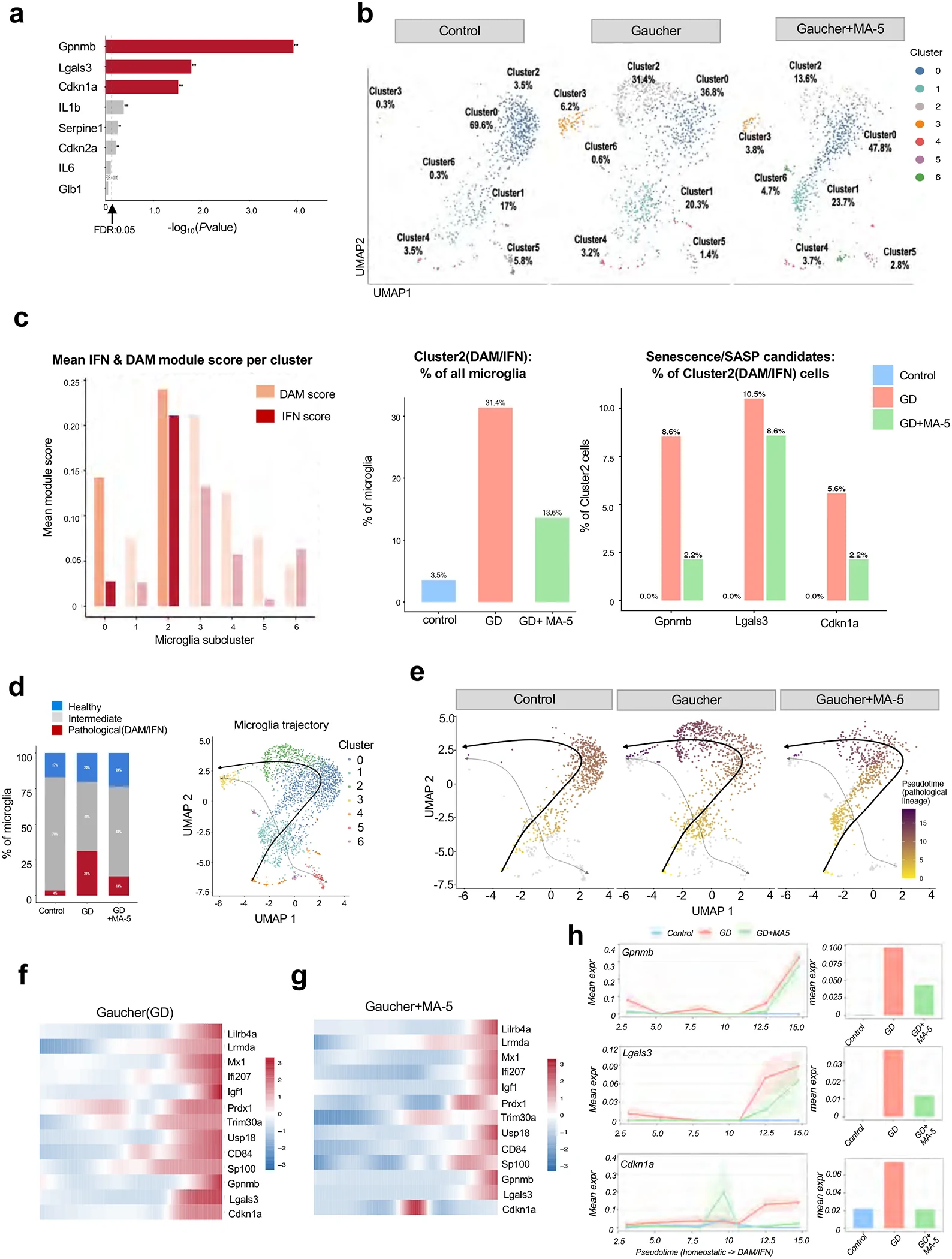
An aberrant microglial differentiation trajectory toward a disease-associated microglia/interferon (DAM/IFN) state, and its partial reversal by MA-5. (a) Statistical significance (−log10 P value, Mann-Whitney U test) of the eight senescence/SASP marker genes from Fig. 3i, restricted to microglia. Dashed line indicates the FDR 0.05 threshold. (b) UMAP embedding of re-clustered microglia (seven unsupervised subclusters, 0–6), split by condition, with the percentage of microglia assigned to each cluster indicated. (c) Left, mean IFN and DAM module score for each microglial subcluster (pooled across conditions), identifying Cluster 0 as the homeostatic state and Cluster 2 as the DAM/IFN state. Middle, percentage of all microglia assigned to Cluster 2, by condition. Right, percentage of Cluster 2 cells expressing Gpnmb, Lgals3, or Cdkn1a, by condition. (d) Left, microglial differentiation trajectory inferred by Slingshot, fitted directly on the UMAP embedding and rooted at the homeostatic cluster; points colored by unsupervised subcluster as in b. Right, branch occupancy (percentage of microglia assigned to the homeostatic, intermediate, or pathological [DAM/IFN] segment of the trajectory), by condition.(e) UMAP embedding as in d (top), split by condition and colored by pseudotime along the pathological lineage. (f) Relative occupancy of early, intermediate, and late pathological pseudotime segments in control, GD, and GD+MA-5 microglia. (g) Heatmaps of 13 pseudotime-associated genes in GD and GD+MA-5 microglia along a shared pseudotime axis, with genes ordered by their peak position in GD. (h) Smoothed expression (tradeSeq; z-scored) of Gpnmb, Lgals3, Cdkn1a, and Irf7 along the pathological pseudotime.

Figure 3 identified Gpnmb, Lgals3, and Cdkn1a as prominent features of the GD-associated pathological microglial state. To determine whether these changes were broadly distributed across microglia or concentrated within specific disease-associated states, we performed higher-resolution re-clustering and trajectory analyses of the microglial population. We first examined the eight aging/senescence-associated genes analyzed in Figure 3 within the microglial population (Figure 4a). Among these, Gpnmb, Lgals3, and Cdkn1a showed the strongest GD-associated differences, with substantially higher −log10(P) values than the other genes examined and Benjamini–Hochberg-adjusted P values below 0.05. Because each condition was represented by a single animal, these cell-level statistical comparisons were interpreted descriptively. These analyses identified Gpnmb, Lgals3, and Cdkn1a as the most prominent disease-associated aging/senescence-related genes among those examined in microglia. Unsupervised re-clustering of microglia, performed independently of the whole-cortex clustering in Figure 2, identified seven transcriptionally distinct subclusters (clusters 0–6), whose relative proportions varied markedly across the three conditions (Figure 4b, **Supplementary Table S1**). Cluster 0 was the predominant microglial population in the control cortex (69.6%) but decreased to 36.8% in GD and partially increased to 47.8% following MA-5 treatment. Conversely, Cluster 2 represented only 3.5% of control microglia but expanded approximately nine-fold to 31.4% in GD before decreasing to 13.6% following MA-5 treatment. Cluster 3 similarly increased from 0.3% in control to 6.2% in GD and decreased to 3.8% following MA-5 treatment. In contrast, Cluster 6 was rare in both control and GD microglia (0.3% and 0.6%, respectively) but increased to 4.7% following MA-5-treatment, consistent with the emergence of an MA-5-associated transcriptional state rather than simple restoration of the control microglial composition.

To define the biological characteristics of these microglial subclusters, we calculated mean DAM and IFN module scores for each cluster using cells pooled across conditions (Figure 4c, left). Cluster 2 showed the highest combined DAM and IFN scores among all subclusters (DAM ≈ 0.24; IFN ≈ 0.21), whereas Cluster 0 showed the lowest scores for both programs, consistent with a relatively homeostatic-like state for Cluster 0 and a disease-associated DAM/IFN state for Cluster 2. Cluster 3 exhibited a high DAM score (≈ 0.21) but a lower IFN score (≈ 0.13), consistent with a damage-associated molecular pattern (DAM)-predominant state distinct from the combined DAM/IFN state of Cluster 2. The proportion of Cluster 2 microglia increased from 3.5% in control to 31.4% in GD and decreased to 13.6% following MA-5 treatment (Figure 4c, middle). We next examined expression of Gpnmb, Lgals3, and Cdkn1a within this DAM/IFN-enriched cluster (Figure 4c, right). In Cluster 2, the proportions of cells expressing Gpnmb, Lgals3, and Cdkn1a were 0% in control and increased to 10.5%, 5.6%, and 8.6%, respectively, in GD. Following MA-5 treatment, these proportions decreased to 8.6%, 2.2%, and 2.2%, respectively. Thus, Gpnmb, Lgals3, and Cdkn1a were preferentially enriched within the Cluster 2 DAM/IFN state rather than broadly expressed across the microglial population. The partial contraction of this disease-associated subpopulation following MA-5 treatment may therefore contribute to the gene-level attenuation observed in Figure 3. Because discrete clustering imposes categorical boundaries on potentially continuous transcriptional states, we next complemented the cluster-based analysis with trajectory inference using Slingshot, with the trajectory rooted in the homeostatic-like microglial region (Figure 4d, **Supplementary Table S5**). This analysis identified a principal cell-state trajectory extending from an early homeostatic-like region through intermediate states toward a late DAM/IFN-enriched pathological state.

Visualization of cells from each condition along the shared trajectory further supported this pattern (Figure 4e). Cells were ordered by pseudotime, representing their relative position along the inferred transition from homeostatic to pathological states. GD microglia were preferentially concentrated toward the later-pseudotime pathological region, whereas control microglia were predominantly distributed at earlier pseudotime positions. GD+MA-5 microglia showed an intermediate distribution, with fewer cells at the pathological end of the trajectory than in untreated GD. We then partitioned cells along this trajectory into early (homeostatic-like), intermediate, and late pathological segments and quantified their relative occupancy by condition (Figure 4f, **Supplementary Table S1**). Control microglia were distributed across these segments at 17%, 79%, and 4%, respectively. In GD, the corresponding proportions shifted to 20%, 48%, and 31%, whereas GD+MA-5 microglia showed an intermediate distribution of 24%, 63%, and 14%. Notably, the trajectory-based estimate of pathological-state occupancy (4% in control, 31% in GD, and 14% in GD+MA-5) closely paralleled the proportion of Cluster 2 cells identified by discrete re-clustering (3.5%, 31.4%, and 13.6%, respectively). Thus, both discrete and trajectory-based analyses showed marked expansion of the pathological microglial state in GD and partial contraction following MA-5 treatment. The homeostatic segment itself was not reduced in GD (17% in control versus 20% in GD). Instead, the largest shift occurred within the intermediate compartment, which decreased from 79% in control to 48% in GD as pathological-state occupancy increased from 4% to 31%. These findings are consistent with a shift of microglia from intermediate toward pathological transcriptional states, rather than depletion of the homeostatic population.

To determine whether MA-5 treatment altered the pseudotemporal expression program of pathological genes or primarily reduced the number of microglia reaching the pathological end of the trajectory, we compared the expression of the 13 pseudotime-associated candidate genes between GD and GD+MA-5 microglia using the same shared pseudotime axis, gene order, and color scale (Figure 4g). Expression was averaged within seven equal-frequency pseudotime bins per condition and jointly z-scored across both conditions, with genes ordered according to the pseudotime position of their peak expression in GD. In GD microglia, the 13 genes showed distinct pseudotemporal expression patterns, with many reaching their highest expression toward the terminal DAM/IFN-associated region of the trajectory. Several genes, including Prdx1, Cd84, and Sp100, also showed increased expression at intermediate pseudotime positions. In GD+MA-5 microglia, the overall late-trajectory organization was retained, but expression intensity was attenuated across much of the gene panel, particularly toward the trajectory terminus. Notably, Cdkn1a and, to a lesser extent, Prdx1 did not simply attenuate in place; instead, their expression peaked at an intermediate pseudotime position following MA-5 treatment and declined toward the trajectory terminus, in contrast to the late-trajectory increase observed in GD. To directly test whether this population-level attenuation reflected reduced expression within individual pathological cells, reduced numbers of cells reaching the pathological state, or both, we examined Gpnmb, Lgals3, and Cdkn1a — the senescence/SASP markers most strongly associated with GD in Figure 3 — together with Irf7, a representative interferon-stimulated gene, at single-cell resolution along the pathological trajectory, alongside each gene’s mean expression across the total microglial population (Figure 4h). At the population level, MA-5 treatment reduced mean expression of all four genes relative to GD (Gpnmb, −55%; Lgals3, −68%; Cdkn1a, −71%; Irf7, −43%), with Cdkn1a returning to a level indistinguishable from control (GD+MA-5, 0.021; control, 0.022). However, the pseudotime-resolved trajectories showed that this population-level rescue was not uniformly explained by reduced expression within individual pathological cells. For Gpnmb and Lgals3, expression in GD and GD+MA-5 converged toward the terminal, DAM/IFN-enriched end of the trajectory (Gpnmb: GD 0.323 versus GD+MA-5 0.276, a 15% difference; Lgals3: GD 0.088 versus GD+MA-5 0.064, a 27% difference, at the latest pseudotime bin), indicating that microglia that progressed to the pathological state retained relatively high expression of these two genes despite MA-5 treatment. In contrast, Cdkn1a and Irf7 showed a substantially larger condition-dependent divergence at late pseudotime (Cdkn1a: GD 0.141 versus GD+MA-5 0.024, an 83% reduction; Irf7: GD 0.221 versus GD+MA-5 0.025, an 89% reduction, at the latest bin), indicating additional within-state suppression of these genes following MA-5 treatment. Together with the reduced occupancy of the pathological trajectory segment following MA-5 treatment, these results indicate that the population-level attenuation of Gpnmb and Lgals3 by MA-5 is explained predominantly by a lower proportion of microglia occupying the terminal pathological state, whereas the attenuation of Cdkn1a and Irf7 additionally reflects within-state suppression in microglia occupying the late pathological region. Together, these findings indicate that MA-5 attenuates the late pathological microglial state through both reduced state occupancy and gene-specific within-state suppression. Because this late pathological state was characterized by prominent aging- and senescence-associated transcriptional features, we next asked whether progression along the GD-associated microglial trajectory was accompanied by accelerated transcriptomic aging, as assessed using transcriptomic aging (tAge)

### MA-5 attenuates GD-associated transcriptomic aging across major brain cell types

**Figure 5.**
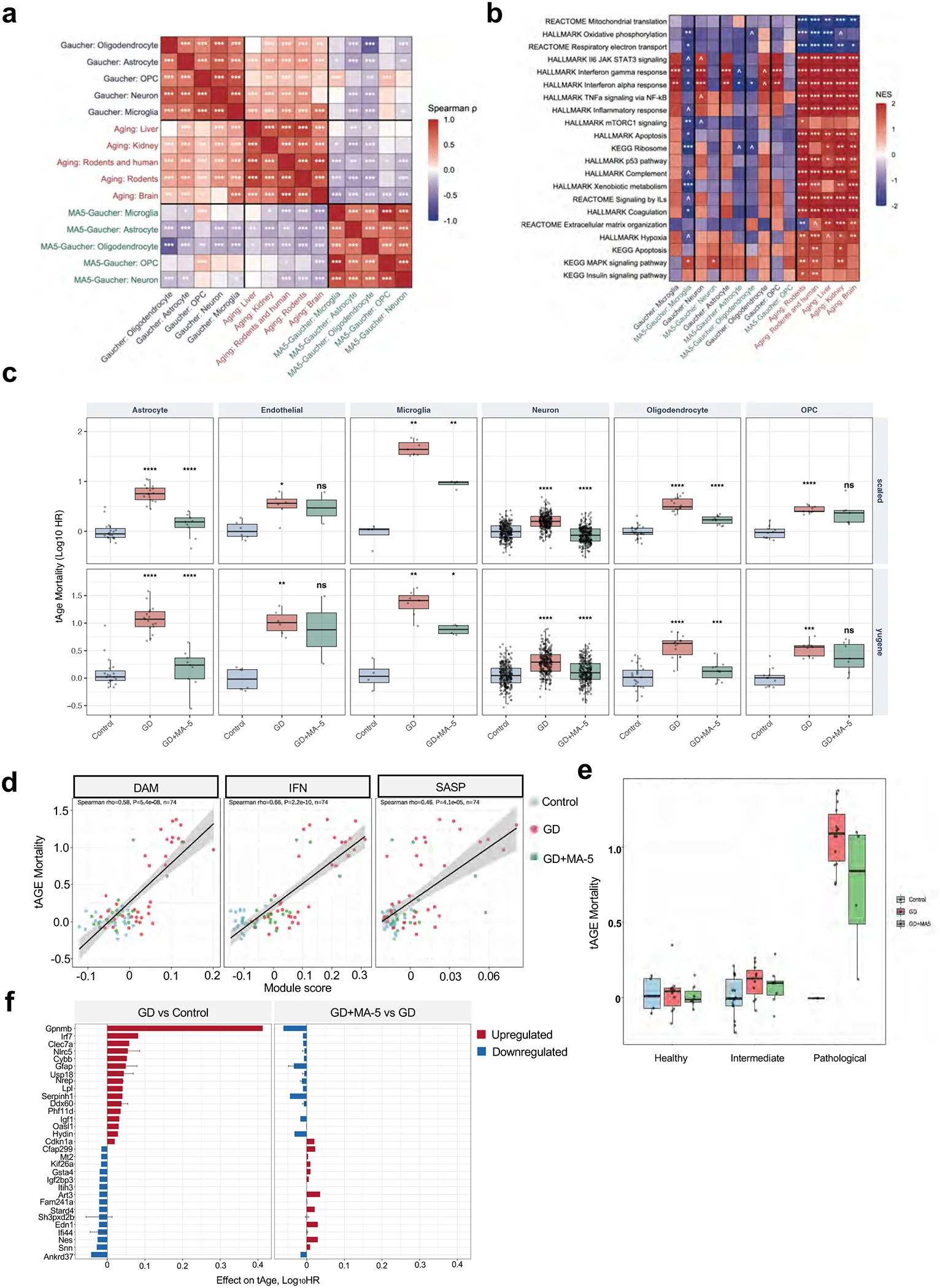
MA-5 attenuates GD-associated transcriptomic aging and pathological microglial aging states. (a) Heatmap showing the correlation of GD-associated transcriptional changes (GD vs. Control) and MA-5-mediated changes (GD+MA-5 vs. GD) with aging and rejuvenation reference signatures derived from multiple tissues, organs, and mammalian species. Positive correlations indicate similarity to aging-associated transcriptional programs, whereas negative correlations indicate similarity to rejuvenation- or reprogramming-associated signatures. (b) Pathway-level analysis of aging-associated transcriptional programs. Heatmaps show the direction and significance of pathway changes in GD and after MA-5 treatment relative to aging and reprogramming reference datasets. Inflammatory and stress-response pathways, including interferon signaling, IL-6–JAK–STAT3 signaling, coagulation, apoptosis, and hypoxia, were elevated in GD and attenuated by MA-5, whereas mitochondrial, metabolic, and oxidative phosphorylation–related pathways showed partial restoration following MA-5 treatment. (c) Transcriptomic age (tAge) was calculated in major brain cell types, including microglia, neurons, astrocytes, oligodendrocytes, and oligodendrocyte precursor cells (OPCs), using the tAge framework. Comparisons among Control, GD, and GD+MA-5 groups are shown. The “scaled” and “yugene” rows show the same mortality clock applied to the same data normalized in two independent ways—persample z-scoring of log_10_ RLE values (“scaled_diff”) or the rank-based YuGene transform (“yugene_diff”), each centered on the Control-group per-gene median—demonstrating that the tAge changes are not an artefact of the normalization method. (d) Correlation of microglial tAge with disease-associated microglia (DAM), interferon (IFN), and senescence-associated secretory phenotype (SASP) module scores. Spearman correlation coefficients are shown. (e) Microglial tAge across healthy, intermediate, and pathological trajectory states in control, GD, and GD+MA-5 conditions, showing increased tAge in the pathological GD state and attenuation by MA-5. (f) Genes contributing most strongly to tAge alterations in the GD brain. The top 25 genes ranked by their contribution to tAge are shown. Genes upregulated in GD and suppressed by MA-5, or downregulated in GD and restored by MA-5, are highlighted. These reciprocal changes shifted the GD transcriptomic profile toward an older state and the MA-5-treated profile toward a younger state. Data are presented as mean – SEM. Statistical significance was determined by one-way ANOVA followed by Tukey’s multiple-comparison test. ^*p*< 0.1, *p< 0.05, **p< 0.01, ***p < 0.001.

We recently developed transcriptomic aging (tAge) clocks that estimate biological age from RNA-seq profiles and capture aging- and mortality-associated transcriptional programs across tissues and species^19,39^. Unlike conventional clocks that primarily predict chronological age, tAge can estimate mortality-associated molecular age, reflecting the biological burden of aging, disease, and lifespan-modulating interventions^19^. We also developed 23 functionally annotated module-specific clocks that quantify aging across individual biological systems^19^. Our previous tAge analyses identified conserved aging-associated transcriptional features, including upregulation of inflammatory, immune, cellular senescence, and pro-mortality programs, and downregulation of tissue-maintenance, mitochondrial, metabolic, and regenerative programs^19,39^. Because chronic neuroinflammation and mitochondrial dysfunction are closely associated with brain aging^3^, we hypothesized that GD may accelerate transcriptomic aging in the brain. To test this hypothesis, we applied the tAge framework to snRNA-seq profiles from major brain cell types, including microglia, neurons, astrocytes, oligodendrocytes, and oligodendrocyte precursor cells (OPCs). GD-associated transcriptional signatures strongly correlated with aging-associated signatures observed across multiple rodent and human tissues (Figure 5a). Subsequently, we performed module analysis to identify the pathways underlying these aging-associated transcriptional changes (Figure 5b). In the GD brain, the IFN-γ response (*p*< 0.001) and IFN-α response (*p*< 0.05) pathways were activated in microglia and changed in the same direction as aging-associated reference signatures. Similar activation of interferon-related pathways was also observed in neurons, astrocytes, and OPCs, indicating an aging-like inflammatory transcriptional state across multiple brain cell types. In contrast, MA-5 treatment markedly suppressed these inflammatory pathways (*p*< 0.05) and shifted the transcriptional profile toward a reprogramming-associated reference signature. Module analysis further showed that MA-5 attenuated multiple aging-associated programs in microglia, including ribosome- and xenobiotic metabolism-related modules (*p*< 0.001), mTORC1 signaling and oxidative phosphorylation modules (*p*< 0.01), and respiratory electron transport, apoptosis, and coagulation modules (*p*< 0.05). IL-6–JAK–STAT3 signaling, inflammatory response, interleukin signaling, and hypoxia-related modules also showed trends toward suppression (*p*< 0.1). These findings indicate that MA-5 attenuates GD-associated transcriptomic aging programs, with particularly pronounced effects in microglia.

To determine whether these module-level changes were accompanied by a measurable shift in overall transcriptomic age, we next directly quantified tAge across individual brain cell types. Application of the tAge framework to the snRNA-seq dataset revealed that transcriptomic age was accelerated in GD mice compared with controls across major brain cell types (microglia, neurons, astrocytes, oligodendrocytes, and oligodendrocyte precursor cells (OPCs)). Notably, MA-5 treatment significantly reduced tAge across these cell populations, indicating a broad reversal of GD-associated transcriptomic aging by MA-5 (Figure 5c, **Supplementary Table S6**). Given the prominent disease-associated transcriptional changes observed in microglia, we next asked whether microglial tAge was linked to specific pathological transcriptional programs. We examined its relationship with DAM, interferon (IFN), and SASP module scores (Figure 5d). Microglial tAge was positively correlated with all three programs, with the strongest association observed for IFN (Spearman ρ = 0.66), followed by DAM (ρ = 0.58) and SASP (ρ = 0.46). These findings indicate that increased tAge is closely associated with disease-associated inflammatory and activation states in microglia. We next examined whether this increase in tAge was preferentially associated with specific microglial states along the disease trajectory. We compared tAge among healthy, intermediate, and pathological trajectory segments (Figure 5e). tAge remained low in healthy and intermediate states but was markedly elevated in the pathological state in GD. Notably, tAge in the pathological state was lower in GD+MA-5 than in untreated GD, consistent with attenuation of the disease-associated aging phenotype by MA-5. Furthermore, to identify individual genes contributing to the GD-associated increase in mortality-related tAge and its attenuation by MA-5, we quantified gene-level effects on tAge (Figure 5f). Gpnmb emerged as the strongest positive contributor to the GD-associated increase in tAge, consistent with its marked induction in GD microglia and enrichment in the late pathological microglial state identified in Figures 3 and 4. Irf7 emerged as the second strongest positive contributor, further linking the interferon-related pathological microglial program to accelerated tAge. Cdkn1a, another prominent GD-associated senescence marker identified in the preceding analyses, also contributed positively to increased tAge. Igf1, which had independently emerged as a prominent late-pseudotime gene in the microglial trajectory analysis (Figure 4g), was likewise identified among the positive contributors to GD-associated tAge. Several of these gene-level contributions shifted in the opposite direction following MA-5 treatment, consistent with attenuation of the GD-associated tAge signature. Notably, Lgals3 was not included in the gene-level tAge analysis because its low expression in control microglia precluded reliable estimation of its contribution relative to the control baseline. Accordingly, its absence from Figure 5f reflects a technical limitation of the gene-level analysis rather than evidence against its association with the GD-related microglial state. These findings link the pathological microglial genes identified in Figures 3 and 4—particularly Gpnmb, Cdkn1a, and Irf7—to the increase in mortality-associated transcriptomic age observed in GD. Together, these findings show that GD is associated with increased tAge across multiple cortical cell populations, whereas MA-5 reduces tAge and partially reverses disease-associated inflammatory and microglial transcriptional programs.

### MA-5 restores mitochondrial homeostasis and reverses tAge in GD patient iPSC-derived microglia

**Figure 6.**
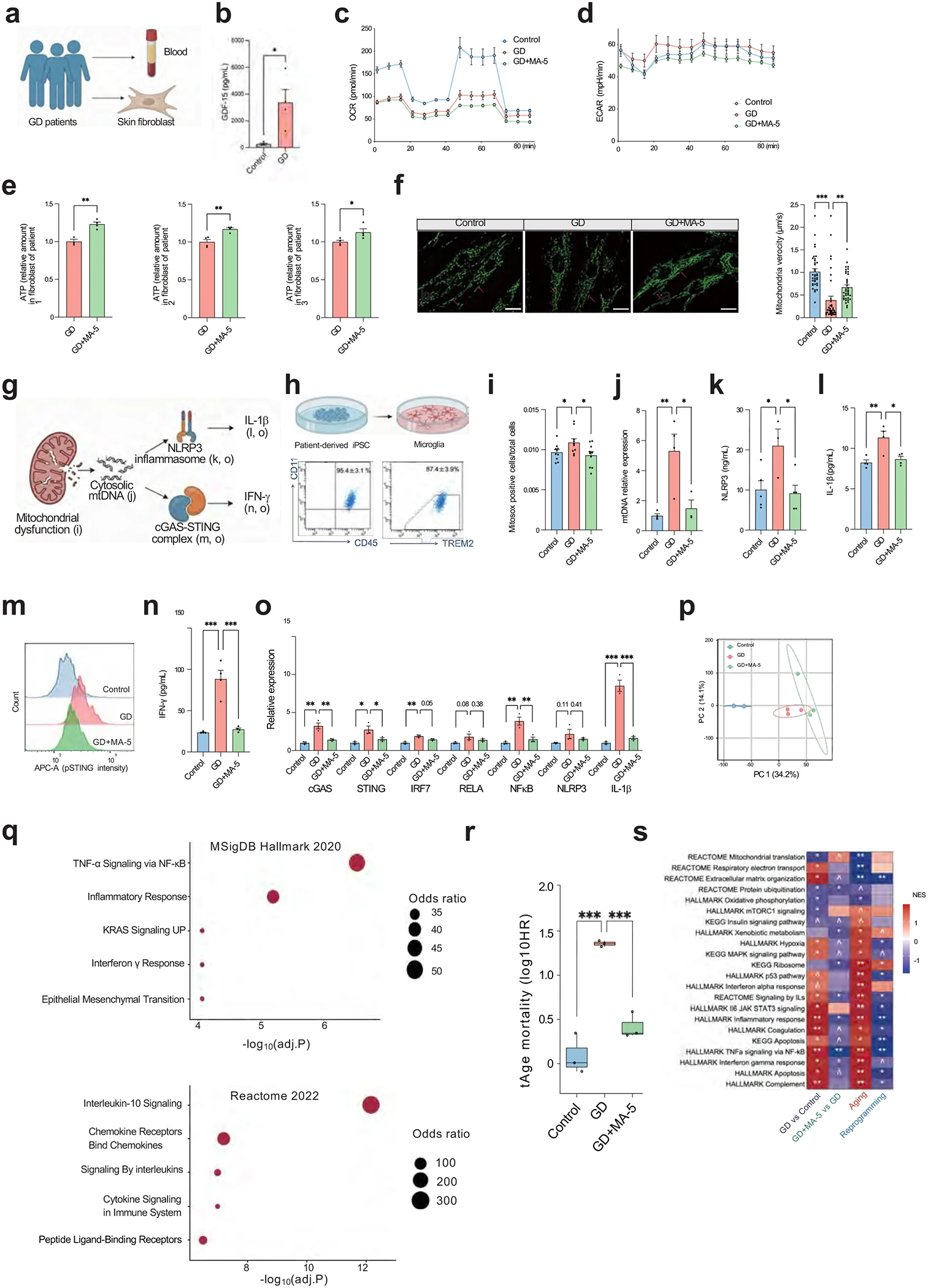
MA-5 suppresses mitochondrial dysfunction–driven innate immune activation and transcriptomic aging in GD patient fibroblasts and iPSC-derived microglia. (a) Schematic of sample collection from GD patients. Blood and skin fibroblasts were obtained for analysis. (b) Serum GDF-15 levels in control and GD patients. N=4 in each group. (c) Oxygen consumption rate (OCR) in control fibroblasts, GD patient fibroblasts, and GD fibroblasts treated with MA-5. (d) Extracellular acidification rate (ECAR) in control fibroblasts, GD patient fibroblasts, and GD fibroblasts treated with MA-5. (e) ATP levels in fibroblasts from three GD patients with and without MA-5 treatment. (f) Representative images and quantification of mitochondrial velocity in control, GD, and GD+MA-5 fibroblasts. Scale bar, 10 μm. (g) Schematic of downstream pathways from mitochondrial dysfunction, including NLRP3 inflammasome and cGAS-STING complex activation. (h) Schematic of differentiation from patient-derived induced pluripotent stem cells (iPSCs) to microglia. Flow cytometry plots showing expression of microglial markers (CD11b, CD45, and TREM2). (i) Proportion of MitoSOX-positive cells in control, GD, and GD+MA-5 microglia. (j) Cytosolic mitochondrial DNA (mtDNA) levels in control, GD, and GD+MA-5 microglia. (k) NLRP3 mRNA expression in control, GD, and GD+MA-5 microglia. (l) IL-1β protein levels in control, GD, and GD+MA-5 microglia. (m) Flow cytometry histograms of phosphorylated STING (pSTING) in control, GD, and GD+MA-5 microglia. (n) IFN-γ protein levels in control, GD, and GD+MA-5 microglia. (o) Relative mRNA expression of cGAS, STING, IRF7, RELA, NFkB, NLRP3, and IL-1β in control, GD, and GD+MA-5 microglia. (p) Principal component analysis (PCA) of transcriptome data from control, GD, and GD+MA-5 iPSC-derived microglia. (q) Pathway enrichment analysis of genes increased in GD and decreased by MA-5 using Hallmark and Reactome databases. (r) Transcriptomic age (tAge) in control, GD, and GD+MA-5 microglia. (s) Gene set enrichment analysis (GSEA) comparing GD vs Control, GD+MA-5 vs GD, aging, and reprogramming datasets. Normalized enrichment scores (NES) are shown. *p<0.05, **p<0.01, ***p<0.001, one-way ANOVA Tukey test.

Our snRNA-seq and tAge analyses suggest that MA-5 attenuates GD-associated neuroinflammation and transcriptomic aging by suppressing microglial inflammatory programs. To assess the translational relevance of the MA-5 effects observed in the GD mouse model and to further investigate the underlying mechanisms, we next examined serum GDF15 levels and mitochondrial activity in patient-derived fibroblasts. GDF15 is a circulating biomarker of mitochondrial dysfunction that is markedly elevated in patients with mitochondrial disorders^18,40,41^. We therefore measured serum GDF15 in patients with GD (Table 1, Figure 6a). The serum GDF15 levels were markedly elevated in all patients with GD, with mean levels more than 15-fold higher than those in the controls, indicating systemic mitochondrial dysfunction (Figure 6b). We next assessed the mitochondrial bioenergetics of the patient fibroblasts by measuring the oxygen consumption rate (OCR) and extracellular acidification rate (ECAR)^16^ (Figure 6c, 6d). GD fibroblasts showed significantly reduced basal and maximal OCR, which was not altered by MA-5 treatment, consistent with previous reports that MA-5 does not alter the proton gradient (Figure 6c and **Supplementary Figure 2a**). ECAR was unchanged in GD fibroblasts but was significantly reduced by MA-5 treatment (Figure 6d, **Supplementary Figure 2b, 2c**). Under these conditions, MA-5 significantly increased intracellular ATP levels in GD fibroblasts from all three patients examined (Figure 6e). These findings suggest that MA-5 enhances ATP production without altering OCR, consistent with previous reports showing that MA-5 preserves mitochondrial structure and improves ATP synthase efficiency^18^. Impaired mitochondrial dynamics have also been reported following reduced oxidative phosphorylation (OXPHOS)^42^, and these abnormalities may be further exacerbated by oxidative stress, increasing cellular vulnerability. Therefore, we next examined mitochondrial dynamics in GD fibroblasts using time-lapse imaging. Mitochondrial mobility was markedly reduced in GD fibroblasts compared to control fibroblasts, whereas MA-5 significantly improved mitochondrial mobility in GD fibroblasts (Figure 6f and **Supplementary Movie 2**). These data further support the mitochondrial impairment in GD.

We further assessed mitochondrial stress–related cell injury using BSO, which induces mtROS and compromises cells with mitochondrial damage^18,41^. In GD fibroblasts, BSO reduced cell viability (**Supplementary Figure 3a**), increased LDH release (**Supplementary Figure 3b**), and elevated ROS levels assessed using DHR123 (**Supplementary Figure 3c**), all of which were ameliorated by MA-5 treatment. Together, these findings demonstrate that mitochondrial dysfunction observed in the GD mouse model is also evident in patients and patient fibroblasts, and that MA-5 improves ATP production and mitochondrial dynamics while protecting GD cells from mitochondrial oxidative stress.

Previous studies have shown that GD-associated mitochondrial dysfunction in microglia promotes mtDNA release into the cytosol, thereby activating inflammatory pathways, such as the NLRP3 inflammasome and cGAS–STING signaling (Figure 6g). To define the mechanisms underlying MA-5-mediated protection against mitochondrial stress and inflammation, we generated microglia from GD patient-derived iPSC lines. Pluripotency, the 1448T>C mutation, GCase deficiency, and reduced GCase activity were confirmed (**Supplementary Figure 4a–d**). The iPSCs were differentiated into microglia, with >90% of the cells expressing CD45, CD11b, and TREM2 (Figure 6h). To mimic inflammatory stress in GD, iPSC-derived microglia were treated with LPS (100 ng/mL). GD microglia showed higher mtROS levels than controls, which were significantly reduced by MA-5 (Figure 6i). Cytosolic mtDNA levels were also significantly increased in GD microglia and restored to control levels following MA-5 treatment (Figure 6j). NLRP3 expression was markedly elevated in GD microglia and was normalized by treatment with MA-5 (Figure 6k). Consistently, IL-1β levels in the culture medium increased in GD microglia and were reduced to control levels by MA-5 treatment (Figure 6l). These findings indicate that MA-5 suppresses mitochondrial stress, cytosolic mtDNA accumulation, NLRP3 inflammasome activation, and IL-1β release in GD patient-derived microglial cells. To further assess the activation of the cGAS–STING pathway, phosphorylated STING (pSTING)–positive cells were quantified using flow cytometry (Figure 6m), and IFN-γ release into the culture medium was measured (Figure 6n). Both pSTING-positive cells and IFN-γ levels were significantly increased in GD iPSC-derived microglia, whereas MA-5 treatment markedly reduced these increases, restoring them to control levels. Real time quantitative polymerase chain reaction confirmed that cGAS–STING pathway genes (cGAS, STING, NF-κB, IRF7, and RELA) and downstream inflammatory mediators (NLRP3 and IL-1β) were elevated in GD microglia and reduced by MA-5 (Figure 6o). These results indicate that the inflammatory activation of the cGAS–STING pathway in GD microglia is suppressed by MA-5.

To characterize the transcriptional changes and tAge responses associated with mitochondrial dysfunction and MA-5 treatment in iPSC-derived GD microglia, we performed RNA-seq analysis. Principal component analysis (PCA) showed a clear separation among the three experimental groups (Figure 6p). To determine whether MA-5 similarly reversed disease-associated transcriptional programs in human iPSC-derived microglia, we performed pathway enrichment analysis of genes upregulated in GD and downregulated by MA-5 (Figure 6q). Hallmark analysis identified prominent enrichment of TNF-α/NF-κB signaling, inflammatory response, and interferon-γ response, whereas Reactome analysis highlighted interleukin, chemokine, and broader cytokine signaling pathways. These findings demonstrate that MA-5 suppresses inflammatory and immune-related transcriptional programs in human iPSC-derived microglia, consistent with the effects observed in mouse microglia. We further examined whether MA-5 also reduced transcriptomic age (tAge) in human iPSC-derived GD microglia, as observed in the GD mouse brain^19^. tAge analysis showed accelerated transcriptomic aging in GD microglia, which was markedly reduced by MA-5 treatment (Figure 6r). We then performed a comparative pathway analysis using aging and reprogramming reference datasets from our tAge framework^19^ (Figure 6s).

Inflammation- and stress-related pathways were broadly activated in iPSC-derived GD microglia compared to controls. These included complement, TNFα signaling via NF-κB, coagulation, and inflammatory response pathways (*p* < 0.01), as well as apoptosis, IFN-γ response, IL-6–JAK–STAT3 signaling, interleukin signaling, MAPK signaling, hypoxia, extracellular matrix organization, and respiratory electron transport pathways (*p* < 0.05). The p53 pathway and IFN-α response also showed trends toward activation (*p* < 0.1). These changes were largely aligned with the aging reference signature and partially overlapped with inflammatory programs observed in GD mouse brain microglia, suggesting that iPSC-derived GD microglia reflect key aspects of the *in vivo* inflammatory state. In the GD+MA-5 versus GD comparison, these pathways were markedly suppressed and shifted toward the reprogramming reference^19^, consistent with the anti-inflammatory trend observed in the GD mouse brain. Conversely, metabolism- and mitochondria-related pathways, including mitochondrial translation, protein ubiquitination, oxidative phosphorylation, and mTORC1 signaling, were downregulated in iPSC-derived GD microglia (*p* < 0.05), broadly consistent with the aging reference observed in GD mouse brain microglia. MA-5 treatment partially restored these metabolic and mitochondrial pathways, suggesting recovery of mitochondrial and cellular homeostatic programs. These findings indicate that iPSC-derived GD microglia exhibit aging-like inflammatory and metabolic dysregulation, that is conserved in the GD mouse model and is partially reversed by MA-5 in a reprogramming-like direction. These results further suggest that MA-5 attenuates GD-associated microglial inflammation by preserving mitochondrial integrity, thereby reducing cytosolic mtDNA accumulation and suppressing NLRP3 inflammasome and cGAS–STING-mediated inflammatory signaling, in association with a reduction in tAge.

### MA-5 restores hepatic function and reverses tAge in GD liver

**Figure 7.**
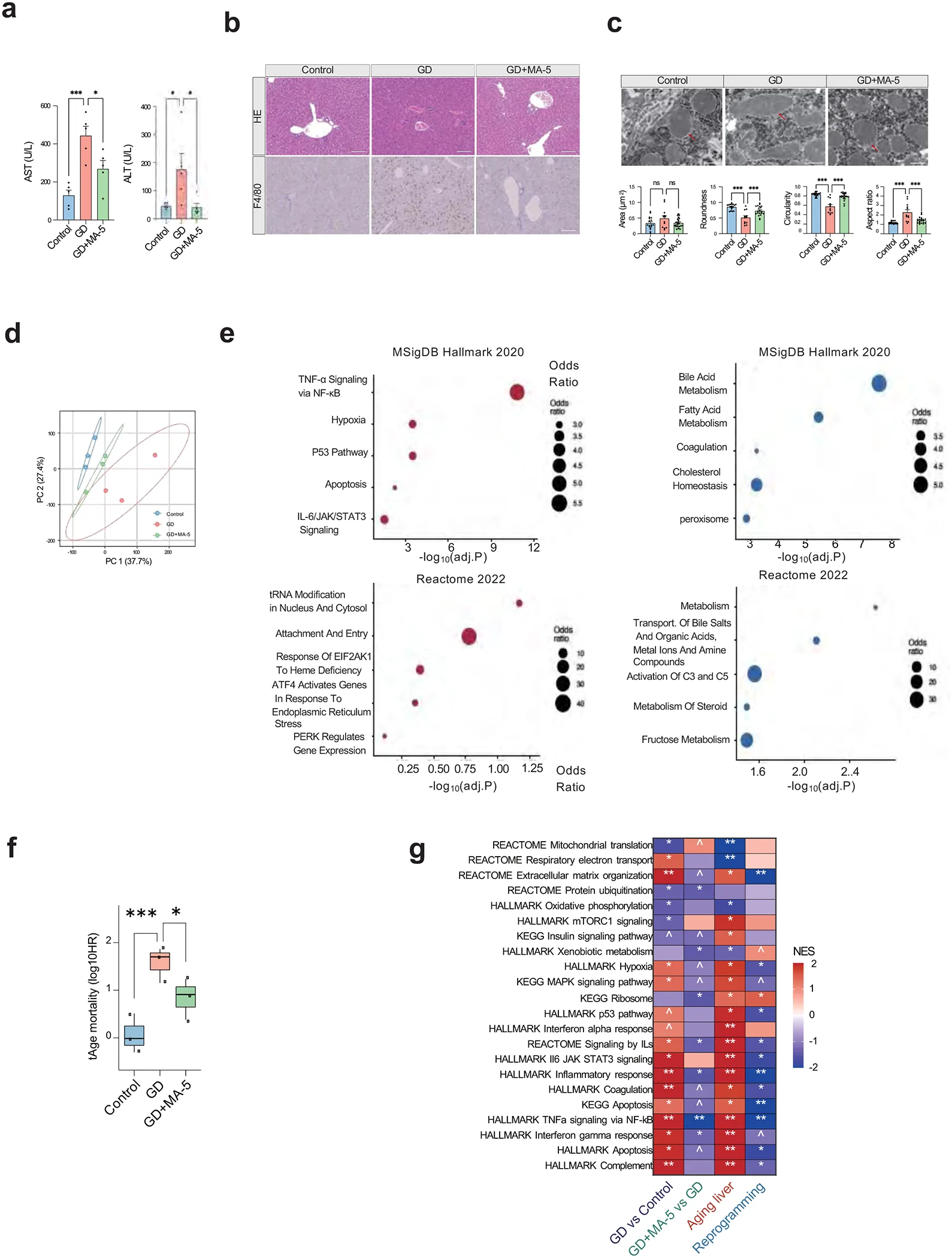
MA-5 ameliorates liver injury and mitochondrial dysfunction and attenuates transcriptomic aging in GD mice. (a) Serum AST and ALT levels in control, GD, and GD+MA-5 mice (Control, n = 5; GD, n= 6; GD+MA-5, n = 5). (b) Representative histological images of liver sections. Upper panel: hematoxylin and eosin (HE) staining. Lower panel: F4/80 immunostaining for macrophages. (c) Electron microscopy images of hepatic mitochondria in control, GD, and GD+MA-5 mice with quantification of morphological parameters (area, roundness, circularity, and aspect ratio). (d) Principal component analysis (PCA) of liver transcriptome data from control, GD, and GD+MA-5 mice. (e) Pathway enrichment analysis of upregulated (left, red) and downregulated (right, blue) genes using Hallmark (top) and Reactome (bottom). Bubble size indicates the odds ratio. (f) Transcriptomic age (tAge) in control, GD, and GD+MA-5 liver samples. (g) Heatmap comparing pathway enrichment across GD vs Control, GD+MA-5 vs GD, aging liver, and reprogramming datasets. Triangles indicate statistical significance; color scale represents enrichment direction. Statistical significance was determined by *p<0.05, **p<0.01, ***p<0.001, one-way ANOVA Tukey test.

Liver involvement is common in GD, particularly in type 1^43^. Elevated AST and ALT levels and steatosis-associated hepatocellular injury have also been reported^44^. In CBE-GD mice, serum AST and ALT levels were markedly elevated compared with controls (AST: 443 – 49.7 vs 130 – 27.2 U/L; ALT: 176 – 56.3 vs 45.4 – 4.58 U/L), and MA-5 significantly reduced these enzyme elevations (AST: 269 – 44.1 U/L; ALT: 40.4 – 14.1 U/L; Figure 7a). Histological analysis also revealed an increased density of hepatocytes and disruption of the normal cord-like architecture in GD^45^ (Figure 7b, upper panel), both of which were ameliorated by MA-5 treatment. F4/80 immunostaining revealed increased macrophage accumulation in the GD liver, consistent with previous reports showing F4/80-positive macrophage/Gaucher-like cells in Gba1 mutant mouse tissues^46^, and macrophage alterations in the liver of a GD mouse model^47^ (Figure 7b, lower panel). Electron microscopy revealed significant alterations in the mitochondrial morphology in GD mice, including changes in roundness, circularity, and aspect ratio, indicative of mitochondrial dysfunction^35^ (Figure 7c). MA-5 treatment restored these abnormal mitochondrial structures. These data suggest that MA-5 not only suppresses neuroinflammation in the GD brain but also improves peripheral disease symptoms, including liver dysfunction, indicating broad therapeutic effects across both central and peripheral compartments.

RNA-seq analysis of liver samples showed that MA-5 treatment shifted the GD transcriptomic profile toward that of control mice, as shown by the PCA (Figure 7d). To characterize transcriptional programs reversed by MA-5 in the liver, we performed pathway enrichment analysis of genes altered in GD and restored in the opposite direction by MA-5 (Figure 7e). Hallmark analysis showed that GD-upregulated genes suppressed by MA-5 were enriched for TNF-α/NF-κB signaling, hypoxia, p53, apoptosis, and IL-6/JAK/STAT3 signaling, whereas GD-downregulated genes restored by MA-5 were enriched for bile acid and fatty acid metabolism, cholesterol homeostasis, and peroxisomal pathways. Reactome analysis similarly highlighted suppression of PERK/ATF4-associated stress responses and restoration of metabolic and bile acid–related pathways. These findings indicate that MA-5 simultaneously attenuates hepatic inflammatory/stress responses and restores metabolic programs impaired in GD. In addition, we calculated tAge in the livers of GD model mice (Figure 7f). Consistent with the findings from the GD mouse brain and human iPSC-derived microglia, GD significantly increased hepatic tAge levels. Importantly, this increase was significantly reversed by MA-5 treatment, suggesting that MA-5 ameliorates aging-associated transcriptional changes in the brain and liver. Because MA-5 reduced the aging hallmark tAge in the brain and iPSC-derived GD microglia, we next examined whether MA-5 also ameliorates age-related transcriptomic alterations in the liver (Figure 7g). By comparative pathway analysis, inflammation- and stress-related pathways were activated in GD livers, and these changes occurred in the same direction as those observed in the aging liver reference. These pathways included extracellular matrix organization, inflammatory response, coagulation, TNF-α signaling via NF-κB, and complement (*p*< 0.01); respiratory electron transport, hypoxia, MAPK signaling pathway, signaling by interleukins, IL-6/JAK/STAT3 signaling, apoptosis, and IFN-γ response (*p*< 0.05); and IFN-α response and the p53 pathway, which showed trends toward activation (*p*< 0.1). Conversely, metabolism- and mitochondria-related pathways, including mitochondrial translation, oxidative phosphorylation, and mTORC1 signaling, were decreased in GD livers (*p* < 0.05). The decrease in mitochondrial translation and oxidative phosphorylation was consistent with that observed in the aging liver reference. In the MA-5-treated group, these pathway changes were largely reversed, showing a pattern similar to that of the reprogramming reference, except for protein ubiquitination. Specifically, inflammation- and stress-related pathways were suppressed, whereas metabolism- and mitochondrial pathways were restored. GD-upregulated signatures, including TNF-α signaling via NF-κB, inflammatory response, and signaling by interleukins, were significantly decreased following MA-5 treatment (*p* < 0.05). Extracellular matrix organization, hypoxia, MAPK signaling pathway, coagulation, and apoptosis also showed trends toward reduction (*p*< 0.1). These findings demonstrate that GD liver exhibits age-like pathway alterations and that MA-5 exerts rejuvenation-like effects similar to those observed during reprogramming. Taken together, these findings demonstrate that GD liver also exhibits age-like pathway alterations characterized by activation of inflammatory and stress-related programs and suppression of metabolic and mitochondrial functions. MA-5 broadly reverses these alterations, exerting rejuvenation-like effects that resemble those observed during reprogramming in the liver.

## Discussion

The present study identifies mitochondrial restoration by MA-5 as a potential strategy for suppressing pathological microglial inflammation, attenuating disease-associated acceleration of transcriptomic age (tAge), and improving neurological dysfunction without reducing lysosomal substrate accumulation. These findings extend the therapeutic significance of MA-5 beyond GD and suggest that restoring mitochondrial integrity may represent a broadly applicable therapeutic target in disorders characterized by chronic neuroinflammation, accelerated cellular aging, or intracellular accumulation of pathogenic materials.

 The first major finding of this study is that MA-5 suppresses pathological microglial activation, a shared pathogenic feature of many neurological diseases. Microglia are essential for maintaining homeostasis in the central nervous system; however, persistent activation can result in excessive production of inflammatory cytokines, reactive oxygen species, and neurotoxic mediators^48–50^. Disease-associated microglia (DAM) and related inflammatory states have been identified not only in GD but also in AD, PD amyotrophic lateral sclerosis, Huntington’s disease, multiple sclerosis, and other neurodegenerative or neuroinflammatory disorders^50^. Despite their diverse initiating causes, these diseases share overlapping microglial programs involving interferon signaling, NF-κB activation, inflammasome activity, and cellular stress. Our single-nucleus transcriptomic analyses showed that GD did not simply induce uniform activation across the microglial population. Rather, it shifted microglia toward a distinct pathological state characterized by DAM, interferon (IFN), NDMG and SASP programs. MA-5 markedly reduced the proportion of microglia occupying this pathological state and suppressed selected disease-associated genes even in cells that had progressed to the late stage of the pathological trajectory. For Gpnmb and Lgals3, the population-level reduction primarily reflected decreased occupancy of the terminal pathological state, whereas Cdkn1a and Irf7 were additionally suppressed within microglia occupying the late pathological region. These observations indicate that MA-5 does not act merely as a nonspecific anti-inflammatory compound. Instead, mitochondrial restoration appears to reshape the distribution and transcriptional identity of microglial states. These effects were also reproduced in patient-derived iPSC microglia, in which MA-5 reduced mitochondrial stress, cytosolic mtDNA accumulation, cGAS–STING signaling, NLRP3 activation, and inflammatory cytokine production, further supporting the translational relevance of this mitochondrial–microglial mechanism.

Together, these findings support a model in which preservation of mitochondrial integrity reduces mtDNA leakage and suppresses multiple interconnected inflammatory pathways, including cGAS–STING, NLRP3, interferon, and NF-κB signaling, rather than targeting a single cytokine or receptor^6,51–53^.

A second major finding is that MA-5 attenuated the disease-associated increase in transcriptomic age (tAge) across multiple cortical cell types, as well as in patient-derived iPSC microglia and the liver. GD increased tAge in neurons, microglia, astrocytes, oligodendrocytes, oligodendrocyte precursor cells, and endothelial cells, indicating that a lysosomal storage disorder can induce a brain-wide aging-like molecular state even at a younger chronological age. In microglia, increased tAge was closely associated with DAM, interferon, and SASP programs and was most pronounced in the pathological segment of the microglial trajectory. MA-5 reduced both the proportion of cells occupying this pathological state and tAge among microglia within that state. Thus, the effects of MA-5 extended beyond the suppression of inflammatory gene expression to the attenuation of a broader aging-like transcriptional state. This pattern is consistent with previous analyses showing that aging tissues exhibit broad activation of interferon, inflammatory, IL-6–JAK–STAT3, TNFα–NF-κB, complement, coagulation, and apoptosis programs, together with suppression of mitochondrial and metabolic programs^54,55^. Mitochondrial bioenergetic failure, altered mitochondrial dynamics, ROS generation, and mtDNA damage have likewise been implicated in chronic inflammation and age-related disease^56^. To our knowledge, these findings provide the first evidence that a pharmacological intervention can attenuate disease-associated acceleration of transcriptomic age across multiple brain cell types *in vivo*. The reduction in tAge, together with improvements in motor function, hearing, survival, mitochondrial integrity, and inflammatory phenotypes, supports the biological relevance of the observed shift toward a younger transcriptomic state^19^. These findings suggest that mitochondrial dysfunction contributes to disease-associated transcriptomic aging through metabolic stress and innate immune activation, and that restoring mitochondrial integrity may simultaneously suppress neuroinflammation and aging-like cellular states. Galectin-3 (Mac-2) provides an additional translational link between inflammation and transcriptomic aging in GD. Galectin-3 and ST2 were initially recognized as markers of cardiac fibrosis and remodeling associated with adverse clinical outcomes^57^. More recently, LGALS3 was identified as a conserved aging- and mortality-associated gene, and circulating galectin-3 was associated with mortality and multimorbidity in the UK Biobank^19^. In the present study, galectin-3 was markedly elevated in the CBE-GD mouse model and was reduced by MA-5. To examine the clinical relevance of this finding, we collected serum samples from an independent cohort of patients with GD (n=7) and matched controls for age, sex, and other relevant clinical factors (n=48). Importantly, serum galectin-3 levels were also elevated in an independent cohort of patients with GD compared with matched controls (**Supplementary Figure 5**), supporting its clinical relevance as a circulating marker of GD-associated inflammation. Elevated circulating galectin-3 has also been reported in inflammation- and fibrosis-associated disorders, including PD^58^, and has been linked to aging, multiple sclerosis, and neurodegeneration^59^. Collectively, these findings support galectin-3 as a potential circulating biomarker of GD-associated inflammation and a candidate marker for monitoring the therapeutic response to MA-5, complementary to GDF15 as a marker of mitochondrial dysfunction. The third major therapeutic implication is that meaningful therapeutic benefits may be achieved without reducing the primary storage material. To date, conventional therapeutic strategies for lysosomal storage disorders have generally aimed to replace the deficient enzyme, reduce substrate synthesis, or promote the clearance of accumulated substrates. In contrast, MA-5 prolonged survival, improved neurological function, ameliorated mitochondrial abnormalities, and attenuated microglia-mediated neuroinflammation and accelerated transcriptomic aging, despite persistent accumulation of GlcCer and Glc-Sph and without substantial normalization of the brain lipidomic profile. Although these conventional substrate-directed approaches remain essential because they target the primary biochemical defect, their efficacy in the central nervous system can be limited by the blood–brain barrier, irreversible tissue injury, incomplete substrate removal, or the persistence of downstream pathological cascades after accumulation has occurred. Our findings suggest a complementary therapeutic principle: disease progression may be attenuated by enhancing cellular resilience and interrupting the downstream mitochondrial–immune cascade, even when the initiating substrate remains present. In this model, substrate accumulation represents the upstream insult, whereas mitochondrial structural failure, mtDNA leakage, innate immune activation, pathological microglial transitions, and accelerated transcriptomic aging function as modifiable mediators of tissue injury. Targeting these downstream processes could provide therapeutic benefit either independently of or in combination with substrate-directed therapies. Such an approach may be relevant not only to lysosomal storage disorders but also to proteinopathies and other accumulation-associated diseases, including AD, PD, and amyotrophic lateral sclerosis, in which complete removal of the accumulated pathogenic material remains difficult.

In conclusion, our findings establish mitochondrial structural integrity as a therapeutically actionable link between intracellular substrate accumulation, pathological microglial activation, and accelerated transcriptomic aging. MA-5 suppressed pathological microglial state transitions and reduced brain-cell tAge while improving disease phenotypes despite persistent glycolipid accumulation. These results introduce the possibility that neurological disease can be modified not only by removing its initiating pathological material but also by preventing its downstream consequences through restoration of mitochondrial integrity. MA-5 may therefore have broad therapeutic potential across neurodegenerative, neuroinflammatory, lysosomal, and other accumulation-associated disorders in which mitochondrial dysfunction serves as a common amplifier of disease progression.

## METHODS

### Materials

[2H5]-MA-5 was synthesized and characterized in our laboratory. LC/MS-grade methanol, acetonitrile, and 2-propanol were obtained from Kanto Chemical Company (Tokyo, Japan), while LC/MS-grade ammonium acetate, acetic acid, and formic acid were purchased from Fujifilm Wako Chemicals (Osaka, Japan). CBE (Conduritol B Epoxide; Cat. #C542) was purchased from Sigma Aldrich (St. Louis, MO).

### Animal Experiments

The animal protocols were approved by the Tohoku University Institutional Animal Care and Use Committee (2020BeA-003–11). This study was conducted in accordance with the Guide for the Care and Use of Laboratory Animals. C57BL/6J mice were obtained from CLEA Japan. Microglial-specific GFP-expressing CX3CR1GFP/+ mice were obtained from Dr. Tomoyuki Furuyasiki. CX3CR1GFP/GFP was crossed with C57BL/6J mice.

Beginning on postnatal day 11, mice were injected intraperitoneally once daily with CBE (50 mg/kg in 50 μL) or PBS^20^. MA-5 was administered orally at 10 mg/kg in 50 μL. The weight of mice was measured daily, before the administration of MA-5. The AST and ALT levels in serum obtained from model mice were measured using a FUJIFILM DRI-CHEM (Cat. #NX500; Fujifilm, Tokyo, Japan) according to the manufacturer’s protocol.

### Hearing function analysis

ABR test was performed as described^60^. Briefly, ABR recording was conducted using a TDT System 3 auditory-evoked potential workstation and BioSigRP software (Tucker-Davis Technologies, Alachua, FL). ABR responses were evoked using bursts of pure tones at frequencies of 4, 8, 12, 16, and 32 kHz. Evoked responses were averaged across 1000 sweeps. The responses were recorded for each stimulus level in 5 dB steps from 100 dB SPL to 10 dB SPL. The ABR threshold was defined as the lowest sound intensity sufficient to elicit at least one peak against the averaged ABR value.

### Quantitation of MA-5 in mouse brain with LC–MS/MS

The MA-5 level in the extracted sample from mouse brain tissue was quantified in the range of 0.02–100 mol/L. A 30-μL aliquot of the calibration standard solution or extracted sample of mouse brain was deproteinized with MeCN–FoOH (100:0.1, v/v) and an internal standard solution of [2H5]-MA-5 solution. The supernatant was concentrated 4-fold by evaporation and reconstitution, and then a 10-μL aliquot of the sample was injected into LC–MS/MS.

LC–MS/MS analysis was performed on an ACQUITY UPLC system (Waters, Milford, MA) coupled to a TSQ Endura (Thermo Fisher Scientific, Waltham, MA) and the chromatographic separation was performed on an ACQUITY UPLC BEH Phenyl column (2.1 × 50 mm, 1.7 μm; Waters). Mobile phases were (A) ammonium acetate (10 mmol/L) and (B) methanol, and a gradient elution from 10% B to 100% B was performed at a flow rate of 500 μL/min. Electrospray ionization was performed in a negative ion mode and MA-5 was detected in the selected reaction monitoring mode at *m/z* 328.1–116.1. The mass transition of [2H5]-MA-5 was set to *m/z* 333.1–121.1. LC–MS/MS control, data acquisition, and data processing were performed using Xcalibur (Thermo Fisher Scientific).

### Motor function analyses

The rotarod test was performed with MK-670 (Muromachi Kikai, Co, Tokyo, Japan). The cylinder speed was set at 20 rpm. A trial ended if the mice fell off the cylinder or remained on the cylinder for more than 1 min. For the grip force test, mice were allowed to grasp a horizontal grid with the four limbs. The bar and grid were connected with a dynamometer (Cat. #GPM-101B; Melquest, Toyama, Japan). Grip strength was measured by slowly pulling the mouse by the tail backward until the mouse released its limbs from the instrument (Melquest). The maximum tension value out of 3 trials was recorded in grams as one measurement.

### Histological analysis

Mouse brains or livers were fixed in 10% neutral buffered formalin and embedded in paraffin. The protocol of staining was based on the paper published previously^61^. Brain sections were stained with anti-galectin-3 antibody (Cat. #14979–1-AP; Proteintech, Rosemont, IL), and anti-m1A antibody (Cat. #D345–3, MBL, Tokyo, Japan), anti-8-OHdG antibody (Cat. #MOG-020P; Japan Institute for the Control of Aging, Shizuoka, Japan), anti-F4/80 antibody (Cat. #123102, BioLegend, San Diego, CA) for liver staining. For BrdU staining, we used BrdU (Cat. #142567; Abcam, Cambridge, UK) and anti-BrdU antibody (Cat. #ab6326; Abcam, Cambridge, UK) following the manufacturer’s protocol.

CX3CR1GFP/+ mice brains were immersed in 4% paraformaldehyde for 24 h and changed to 30% sucrose for 24 h. After the brains were rapidly frozen in OCT compound (Sakura Finetek, Osaka, Japan), coronal brain sections of 30 μm thickness were made using a cryostat (Carl Zeiss MicroImaging GmbH, Jena, Germany). Images were captured and processed using a BZ-Z810 fluorescence microscope (Keyence, Osaka, Japan). Galectin-3-positive area and m1A positive area were calculated by using BZ-H3A software (Keyence). 8-OHdG or BrdU positive cells were counted by a blinded observer. For measuring the density of activated microglia in CX3CR1GFP/+ mice brains, activated microglia were identified by morphology (round vs stellate) and counted by an experimenter blinded to the samples using ImageJ.

### Lipidomics by UHPLC-MS/MS

Brain lipids were extracted using a modified Bligh-Dyer method^62^ with stable isotope-labeled internal standards (UltimateSPLASH ONE, Avanti Research, Alabaster, AL). UHPLC-MS/MS analysis was performed on a Vanquish UHPLC system coupled to a QExactive Orbitrap mass spectrometer (Thermo Fisher Scientific) as previously described^63,64^.

### DESI-cIMS-MSI

DESI-cIMS-MSI was performed using a Waters SELECT SERIES CyclicIMS system equipped with a DESI XS source (Waters Corp., Milford, MA). GlcCer and GalCer were separated by cyclic ion mobility (20 passes, *m/z* 726.4). The optical image was taken by a camera and imported to the HDImaging software version 1.6. (Waters Corp.) for the imaging acquisitions. Three brain areas were acquired by DESI-cIMS-MSI on a 75 μm × 75 μm pixel size with a raster rate of 150 μm/s. All raw files were imported to the same software for further data processing to create the visualized image of *m/z* 726.4.

### RNA sequencing (RNA-seq)

Bulk RNA-seq was performed on human iPSC-derived microglia, mouse liver tissue, and hepatocytes. For iPSC-derived microglia, total RNA was extracted using a low-input kit (NucleoSpin RNA XS Plus, Macherey-Nagel, D ren, Germany) and libraries were prepared with the SEQuoia Complete Stranded RNA Library Prep Kit (Bio-Rad, Hercules, CA). For mouse liver and iPSC-derived hepatocytes, libraries were prepared using QIAseq Stranded RNA Library Kits (QIAGEN, Venlo, Netherlands) as previously reported^61^. All samples were sequenced on an Illumina NovaSeq platform (150 bp paired-end) at Haplo Pharma (Sendai, Japan). Reads were aligned to GRCh38 (human) or GRCm39 (mouse) using STAR (v2.7.10a). For microglia samples, quantification was performed with RSEM (v1.3.3) after quality trimming by Cutadapt (v4.4); for liver and hepatocyte samples, reads were quality-filtered with Trimmomatic (v0.39) and counted with StringTie (v2.2.0). Differential expression analysis was performed using edgeR (v4.0.16) with trimmed mean of M-values (TMM) normalization.

### Nucleus isolation from mice

Nuclei were isolated from mice brain using the Minute Single Nucleus Isolation Kit for Neuronal Tissues/Cells (Cat. #BN-020, Invent Biotechnologies, Inc., Plymouth, MN) according to the manufacturer’s instructions. The isolated nuclei were checked for integrity and quantity using a microscope before proceeding to snRNA-seq.

### Single-nucleus RNA sequencing (snRNA-seq)

A suspension of nuclei from the brains of P22 mice containing approximately 1 × 10^4 nuclei cells was loaded into a Chromium Next GEM Chip K Single Cell Kit (Cat. #PN-1000287, 10x Genomics, Pleasanton, CA), and a Chromium Controller was used for barcoding and cDNA synthesis. A Chromium Next GEM Single Cell 5’ Library & Gel Bead Kit v2 (Cat. #PN-1000265, 10x Genomics) was used to amplify the cDNA and construct the cDNA libraries. RNA sequencing was performed by DNAFORM (Yokohama, Kanagawa, Japan). For three distinct datasets - Control, GD, and GD +MA-5, the median sequencing depth was 18,547, 17,563, and 20,815 reads per cell, with 93.0%, 93.3%, and 93.7% of the reads confidently mapping to the genome and 13,014, 14,385, and 11,570 nuclei captured, respectively. Sample processing and analysis were performed using Seurat (v5.3.1). Cells with mitochondrial content > 10%, < 200 genes, or > 4,000 genes were excluded as dying cells, empty droplets, or doublets, respectively. Cluster identity was determined by UMAP based on differentially expressed genes and cross-referenced with the Human BioMolecular Atlas Program single-cell reference data (https://azimuth.hubmapconsortium.org/).

Cell identity gene expression scores were assigned using the AddModuleScore function and visualized using FeaturePlot_scCustom from scCustomize (v0.7.0). Approximately 8,000 nuclei were analyzed per group, enabling single-cell-level transcriptional profiling. The disease-associated microglia (DAM) score^3^, microglia activation score^38^, IFN activation score^3^, NDMG score^3^, and senescence-associated secretory phenotype (SASP) score^65^ were computed and shown as heatmaps on UMAP plots.

In the microglial dataset, contaminating non-microglial cells were filtered out based on the microglial score, with particular attention to removing Mrc1+ macrophages. The remaining cells were subsetted and reclustered at a resolution of 0.5. Differential gene expression analysis was performed using Seurat’s FindMarkers function with the MAST test (v1.22.0), implementing a two-part hurdle model with Bonferroni correction. Data were normalized using NormalizeData (LogNormalize). Significantly changed genes (P<0.05) were analyzed by gene set enrichment analysis using Enrichr. For subclustering analyses, genes with log_2_ fold change ≥ 0.5 by Wilcoxon rank-sum test were selected for downstream pathway analyses.

### snRNA-seq data processing and cell-type-level analysis

All downstream single-nucleus RNA-seq analyses were performed in R (v4.4.2) using Seurat (v5.3.1) on the integrated and annotated Seurat object described above. Unless otherwise specified, analyses focused on six major parenchymal cortical cell populations: neurons, oligodendrocytes, astrocytes, oligodendrocyte precursor cells (OPCs), microglia, and endothelial cells. The macrophage population was excluded from cell-type-resolved analyses because it represented a distinct non-parenchymal population. Cell-type composition was calculated as the percentage of analyzed nuclei assigned to each of the six major cell types within each condition. For fine-grained cluster-level abundance analysis, the proportion of nuclei assigned to each unsupervised cluster was calculated separately for Control, GD, and GD+MA-5 conditions. Log2 fold changes in cluster abundance were calculated for GD versus Control, GD+MA-5 versus GD, and GD+MA-5 versus Control using a pseudocount of 0.05. A cluster was descriptively considered to show MA-5-associated reversal when the direction of the GD-versus-Control change was reversed in GD+MA-5 versus GD and the magnitude of the remaining difference from Control was reduced.

For each major cell type, transcriptome-wide perturbation was quantified as the mean absolute log2 fold change across the top 3,000 variable genes identified using Seurat FindVariableFeatures with the variance-stabilizing transformation method. Pseudobulk expression was calculated as counts per 10,000, with a pseudocount of 0.1, and perturbation scores were calculated separately for GD versus Control and GD+MA-5 versus Control. Because each condition was represented by a single animal, these scores were interpreted descriptively as measures of transcriptome-wide deviation from the corresponding control population rather than as inferential estimates of between-animal effects

### Pathway-level analysis of snRNA-seq data

Literature-derived gene signatures representing DAM, IFN response, NDMG and SASP were evaluated using Seurat AddModuleScore. Gene lists used for each signature are provided in **Supplementary Table S4**. Signature scores were visualized on the shared UMAP embedding and compared across experimental conditions and major cortical cell populations. To characterize pathway-level transcriptional alterations, seven curated gene sets representing lysosomal, autophagic, mitochondrial oxidative phosphorylation (OXPHOS), oxidative-stress, integrated stress response (ISR), inflammatory, and senescence/SASP programs were analyzed. Per-cell module scores were calculated using AddModuleScore, and mean module scores were summarized for each condition and major cell type. Disease-associated changes were expressed relative to the corresponding cell-type-specific Control mean for GD and GD+MA-5. The genes included in each curated pathway module are provided in **Supplementary Table S3**.

### Transcription factor activity inference

Transcription factor (TF) activity was inferred from pseudobulk expression profiles using decoupleR and curated mouse DoRothEA regulons with confidence levels A–C. Mean CP10K expression was calculated for each cell type–condition combination and log1p-transformed. Cell type–condition combinations containing fewer than five cells were excluded. TF activity was estimated using the univariate linear model implemented in decoupleR. To distinguish condition-associated TF activity from baseline differences among cell types, the TF activity score of the corresponding Control population was subtracted from GD and GD+MA-5 scores within each cell type. TFs were ranked according to the maximum absolute deviation from the cell-type-specific Control baseline across GD and GD+MA-5 conditions. The resulting relative TF activity profiles were used to identify condition-associated regulatory programs, including the ISGF3 components Stat1, Stat2, and Irf9 and the inflammatory regulator Nfkb1

### Pseudotime-associated gene-expression analysis

Gene-expression dynamics along the pathological microglial trajectory were modeled using tradeSeq. Negative-binomial generalized additive models were fitted for each gene as a function of pathological-lineage pseudotime and Slingshot lineage weights using fitGAM with six knots. Associations between gene expression and pseudotime were assessed using associationTest with a Wald test, and P values were adjusted for multiple testing using the Benjamini–Hochberg procedure. For direct comparison of pseudotemporal expression patterns between GD and GD+MA-5 microglia, cells were divided into seven equal-frequency pseudotime bins. Bin boundaries were defined using the pooled pseudotime distribution across conditions, and mean expression was calculated separately within each condition and bin; bins containing fewer than three cells for a given condition were excluded. Expression profiles were jointly z-scored across GD and GD+MA-5 so that relative expression intensity could be directly compared between conditions. Genes were ordered according to the pseudotime position of their peak expression in GD. For heatmap visualization, the seven-bin expression profiles were interpolated using a monotone Fritsch–Carlson Hermite spline. Interpolation was used solely for visualization and did not alter the underlying binned expression values. For Gpnmb, Lgals3, Cdkn1a, and Irf7, mean expression and standard error of the mean (SEM) were additionally calculated within the same seven pseudotime bins for each condition. These pseudotime-resolved expression profiles were compared with mean expression calculated across the total microglial population irrespective of pseudotime.

### Transcriptomic age (tAge) analyses

To estimate transcriptomic age (tAge), we applied rodent multi-tissue chronological and mortality transcriptomic clocks based on the Bayesian Ridge algorithm to non-overlapping metacells computed separately for each animal and brain cell type^19,39^. Non-overlapping random subsets of cells per animal and cell type were pooled to achieve a target coverage of 250,000 reads per metacell; metacells with fewer than 100,000 reads were excluded. Within each cell type, metacell count data were filtered to retain genes with at least 7 reads in ≥20% of metacells, log-transformed, normalized using Relative Log Expression (RLE), and scaled. Scaled log-expression profiles were centered relative to the median profiles of control mice. Missing values for clock genes were imputed using precalculated average expression values. tAge predictions were compared between groups within each cell type using a mixed-effects model via the metafor R package, and p-values were adjusted using the Benjamini–Hochberg procedure.

### ELISA

The levels of GDF-15 in human serum were determined using a Quantikine Human GDF-15 ELISA Kit (Cat. # DGD150; R&D Systems, Minneapolis, MN). The Human NLRP3 ELISA Kit was used to measure the levels of NLRP3 in microglia (Cat. #ab274401; Abcam, Cambridge, UK). The levels of IL-1β in cell culture supernatants were determined using the Quantikine Human IL-1β ELISA Kit (Cat. #DLB50; R&D Systems), and IFN-γ levels were determined using the AuthentiKine Human IFN-gamma ELISA Kit (Cat. #KE00146; Proteintech, Rosemont, IL). The level of galectin-3 in murine serum was measured with Mouse Galectin 3 ELISA Kit (Cat. #ab203369, Abcam).

### GCase Activity Assay

GCase activity assay was performed as described^66^. Briefly, fibroblast lysates were incubated at 37 °C with the substrate solution in 0.1 M citrate buffer, pH 5.2, supplemented with sodium taurocholate (0.8% w/v) for 1 h. The reactions were terminated by adding 0.2 μL of 0.2 M glycine sodium hydroxide buffer (pH 10.7). The liberated 4-methylumbelliferone (Cat. #M1381; Sigma Aldrich, St. Louis, MO) was measured spectrophotometrically using SpectraMax M2e (Molecular Devices, San Jose, CA) (excitation wavelength: 340 nm; emission: 460 nm).

### Cell viability assay and LDH level assay

Cell viability assay and LDH level assay were performed as previously reported^16,17,41^. BSO (L-buthionine-[S,R]-sulfoximine) was purchased from Sigma Aldrich (Cat. #B2640；St. Louis, MO). GD fibroblasts were cultured in normal growth medium until semi-confluent and then plated at 3 × 10^3 cells in 0.2 mL assay medium per well of a 96-well flat-bottom culture plate. After 24-hr incubation, BSO at 100 μM or dimethyl sulfoxide (DMSO, Cat. #13445–74, Nacalai Tesque) at 0.1% were added, and the cells were cultured for another 24 h. After each compound was applied at the indicated final concentrations, fibroblasts were cultured for 48 h, and cell viability was measured by Cell Count Regent SF (Cat. #07553–15, Nacalai Tesque). The LDH level was measured by cell counting using an LDH cytotoxicity detection kit (Cat. #MK401; Takara, Shiga, Japan).

### Measurement of mitochondrial function by a flux analyzer

The oxygen consumption rate (OCR) and extracellular acidification rate (ECAR) of fibroblasts from GD patients and normal volunteers were measured using XFe24 (Agilent Technologies, Santa Clara, CA). Briefly, fibroblasts were cultured in assay medium (70 mM sucrose, 220 mM mannitol, 10 mM KH_2_PO_4_, 5 mM MgCl_2_, 2 mM HEPES, 1.0 mM EGTA and 0.2% [w/v] fatty acid-free BSA, pH 7.2) without CO_2_ for 60 min. Then, after equilibration, the three respiration rates were measured in fibroblasts following injections of four inhibitors (1 μM oligomycin, 1 μM carbonyl cyanide-p-trifluoromethoxyphenylhydrazone (FCCP), 1 μM rotenone and antimycin) of mitochondrial oxidative phosphorylation (OXPHOS). Each reagent was obtained from Sigma Aldrich (Cat. #75351, Cat. #C2920, Cat. #557368, Cat. #A8674, respectively)

### Measurement of ATP production after treatment with DMSO or MA-5

Fibroblasts from GD patients were cultured in 96-well plates at a density of 3.0 × 10^3 cells per well (n = 4). The ATP level was measured 6 h after DMSO or MA-5 treatment (10 μM) by an ATP measurement kit (Cat. #CA2–10; Toyo Ink, Tokyo, Japan).

### Live imaging of mitochondrial dynamics

Fibroblasts of GD patients and normal volunteers were cultured in dishes and stained with Mitotracker Green (Cat. #M7514; Invitrogen, Waltham, MA). GFP images were taken every 5 seconds for 5 minutes with a KEYENCE BZ-X810 all-in-one fluorescence microscope equipped with a time-lapse tracking system (Keyence). The movies were analyzed with the video editing analysis software VW-H2MA (Keyence)^41^.

### iPSC reprogramming and culture

Informed consent was obtained from the donor from whom iPSC was derived. GD patient-derived fibroblasts were reprogrammed into iPSCs at Unitech Inc. On day zero, 5 × 10^3 cells/cm^2 fibroblast cells were seeded with fibroblast medium described in the prior section, and transfected by adding vectors from NHDF Nucleofector Kit, Epi5 Episomal iPSC Reprogramming Kit (Cat. #A15960; Invitrogen, Waltham). After 24 h, the medium was replaced with N2B27 medium. After the transfection, colonies were picked into separate wells. Pluripotency was confirmed by alkaline phosphatase staining. After the iPSC cell line was established, it was passaged as colonies with ReLeSR (Cat. #100–0484; StemCell Technologies, Vancouver, BC, CA) for 4 min at 37 °C and replated into plates coated with Geltrex (Cat. # A1569601; Life Technologies, Waltham, MA). iPSCs were cultured in mTeSR plus (Cat. #100–0276; StemCell technologies) including 10μM Y-27632 dihydrochloride (Cat. #sc-216067, Santa Cruz Biotechnology, Dallas, TX) for the first 24 h after passage and then cultured in mTeSR1 without Y-27632 dihydrochloride.

### Immunostaining of GD patient iPSC

The immunostaining was performed as previously described^67^. Briefly, patient-derived iPSCs were fixed with 4% PFA/PBS at 4 °C for 15 min after a brief wash with PBS. Fixed cells were washed with PBS and then blocked with PBST (PBS/0.4% Triton X-100)/5% normal donkey serum (Cat. #017-000-121, Jackson ImmunoResearch Laboratories, West Grove, PA) for 1 h at room temperature. Primary antibodies were diluted in blocking solution and incubated with the fixed cells for 3 h at room temperature. After washing with PBST, secondary antibodies were incubated for 30 min at room temperature. Fluorescent images were captured by BZ-X800 (Keyence).

### Differentiation into microglia and its treatment

Microglia were differentiated using a commercially available differentiation kit based on a previously published protocol^68^. Briefly, iPSCs were differentiated into hematopoietic cells using the STEMdiff Hematopoietic Kit (Cat. #05310; StemCell Technologies, Vancouver, Canada). Floating hematopoietic cells were then collected and plated in a 6-well plate coated with geltrex at 1 × 10^5 cells/well in STEMdiff microglia differentiation medium (Cat. #100–0019; StemCell Technologies) for 24 days, followed by passage in a 6-well plate coated with matrigel at 1 × 10^6 cells/well in STEMdiff microglia maturation medium (#100–0021; StemCell Technologies) for at least 4 days. Microglia were seeded into 48-well plates or 96-well plates coated with Geltrex at a density of 1 10^5 cells/well and then treated with 100 ng/mL LPS (Cat. #00-4976-93; Thermo Fisher Scientific) for 3 h, and 1 μM MA-5 for 24 h.

### Flow cytometry

iPSC-derived microglia were fixed in PBS containing 4% paraformaldehyde and then blocked and stained with FITC anti-CD11b antibody (1:100; Cat. #101207; Biolegend, San Diego, CA) and PE anti-CD45 antibody (Biolegend, 301329, 1:100) and anti-TREM2 antibody (1:100; Cat. #MAB17291-SP; R&D Systems) in PBS containing 1% FBS for 45 min.

### Mitochondrial superoxide analysis

To determine mitochondrial ROS production of iPSC-derived microglia, we used MitoSOX Red mitochondrial superoxide indicator (Cat. #M36008; Invitrogen) as described. Briefly, cells were treated with 5 μM MitoSOX and the nuclear staining dye Hoechst-33342 (Sigma Aldrich). Fluorescence was recorded for (excitation (Ex) 510 nm and emission (Em) 580 nm) and normalized to Hoechst-33342 (Ex 350 nm and Em 461 nm) in a SpectraMax M2e (Molecular Devices, San Jose, CA).

### Measurement of mtDNA in cytosol of microglia

Microglial cell lysates were dissolved in Digitonin (Cat. #19390–04; Nacalai Tesque) buffer (100 μg/mL), incubated at 37 °C for 5 minutes, and then centrifuged at 1000 × g for 5 minutes. The supernatant was used as the cytosolic fraction. DNA contained in the cytosolic fraction was extracted using NucleoSpin DNA RapidLyse (Cat. #740100.10; Macherey-Nagel, D ren, Germany). The copy number of mtDNA was normalized to that of nuclear DNA as the ratio of DNA encoding cytochrome c oxidase I to nuclear DNA encoding 18S ribosomal RNA. The primers used are listed in **Supplementary Table S7**.

### Reverse transcription and quantitative PCR

Total RNA was extracted using RNeasy Mini Plus (Qiagen). cDNA was synthesized using a ReverTra Ace qPCR RT Kit (Cat. #FSQ-101; TOYOBO). Quantitative real-time PCR was performed using SYBR Green PCR reagents according to the manufacturer’s instructions on a StepOnePlus^™^ Real Time PCR System (Thermo Fisher Scientific). The primers used are listed in **Supplementary Table S7**. Relative mRNA expression was calculated using the ΔΔCt method and normalized to GAPDH.

### Transmission electron microscopy

Mouse brains were fixed in 2% formaldehyde and 2.5% glutaraldehyde in PBS overnight. The tissues were then washed with PBS and post-fixed in 2% osmium tetroxide for 2 h. All samples were washed twice in PBS, dehydrated in ascending grades of ethanol and embedded in TAAB Epon 812 (TAAB, Reading, UK). Serial sections (70 nm in thickness) were cut on an Ultracut UCT ultramicrotome (Leica Microsystems, Wetzlar, Germany). After being stained with uranyl acetate and lead citrate, they were examined on a JEM-1400 transmission electron microscope (JEOL, Tokyo, Japan) operated at 80 kV. Digital images were collected by a JEOL charge-coupled device (CCD) camera.

### DNA sequencing

Genomic DNA was isolated from fresh iPSC samples by Cell Lysis Solution (Qiagen, Hilden, Germany) and Protein Precipitation Solution (Qiagen) according to the manufacturer’s instructions. The primers used are listed in **Supplementary Table S7**. PCR was performed using KOD FX Neo (Cat. #KFX-201; Toyobo, Osaka, Japan) in a Gene Amp PCR System 9700 (Thermo Fisher Scientific) under the following conditions: 1 cycle of 94 °C for 2 min and 35 cycles of 10 s at 98 °C, 30 s at 50 °C and 30 s at 68 °C. PCR products were sequenced by the Sanger method on an automated DNA sequencer (3500xL Genetic Analyzer, Applied Biosystems, Thermo Fisher Scientific) using BigDye Xterminator Purification Kit (Thermo Fisher Scientific), according to the manufacturer’s instructions.

### Software and packages

All computational analyses were performed using R (v4.4.2) and Python (v3.14). Single-cell RNA sequencing was analyzed with Seurat (v5.3.1) and differential expression testing with MAST (v1.22.0). Pathway and gene set enrichment analyses were performed using fgsea (v1.34.2), msigdbr (v25.1.1), and enrichR (v3.2). A complete list of all software packages and versions is provided in the Key Resources Table.

### Statistical analysis

Data are presented as mean – standard error. Statistical significance was determined using Student’s or Welch’s *t*-test or one-way ANOVA with Tukey’s post hoc test. Statistical threshold was set at *p* < 0.05. GraphPad Prism 10 (Version 10.3.0, GraphPad Software, Boston, MA) was used for statistical analyses.

## Supplementary Material

Figure.S1 to S5

Supplementary Table S1-S7

Supplementary Movies 1 and 2

**Supplementary Figure 1** The CBE model selectively recapitulates the conserved microglial inflammatory program of genetic GD.(a) Scatter plot comparing differential gene expression changes in CBE model microglia and GBA knockout microglia from the scRNA-seq dataset of Boddupalli et al.^37^. Genes with adjusted P < 0.05 in either dataset were included (n = 1,158). A strong positive correlation was observed between the two datasets (Pearson r = 0.742), indicating substantial concordance of microglial transcriptional changes across models. Representative shared upregulated genes included interferon- and disease-associated microglial genes such as Gpnmb, Iigp1, Oasl1, and Irf7. (b) Scatter plot comparing differential gene expression changes in CBE pseudobulk microglia and sorted microglia from the GbaΔNes model reported by Shimizu et al.^38^ Genes with adjusted *P* < 0.05 in either dataset were included (n = 3,430). The two datasets showed a similarly strong positive correlation (Pearson r = 0.741), with concordant upregulation of inflammatory genes including Cxcl10, Irf7, and Ifi44.

**Supplementary Figure 2** Mitochondrial dysfunction and MA-5 response in GD patient fibroblasts. (a) Mitochondrial function analysis in Patient 1 fibroblasts measured by Seahorse flux analyzer. Parameters include basal respiration, maximum respiration, ATP production, proton leak, non-mitochondrial respiration, and ECAR (extracellular acidification rate) comparing control (blue), GD (red), and GD+MA-5 (green). Statistical significance: * *p* <0.05, ** *p* <0.01, *** *p* <0.001, ns=not significant. (b) Mitochondrial function in Patient 2 fibroblasts. Top: Time-course measurements of OCR (oxygen consumption rate) and ECAR comparing control (black) and Patient 2 (blue). Bottom: Quantified metabolic parameters showing significant impairment in GD cells (***p<0.001). (c) Mitochondrial function in Patient 3 fibroblasts. Top: Time-course measurements of OCR and ECAR. Bottom: Quantified metabolic parameters showing significant impairment in most parameters (***p <0.001). Statistical analysis: Two groups compared by Student’s t-test; three groups compared by one-way ANOVA with Tukey’s test.

**Supplementary Figure 3** Oxidative stress resistance in GD patient-derived fibroblasts. (a) Cell viability in fibroblasts from three GD patients with and without BSO (L-buthionine-(S,R)-sulfoximine) treatment and MA-5. (b) LDH (lactate dehydrogenase) release in fibroblasts from three GD patients with and without BSO treatment and MA-5. (c) Representative fluorescence images and quantification of DHR123-positive area in GD patient fibroblasts with BSO and MA-5 treatment. Statistical significance was determined by *p<0.05, **p<0.01, ***p<0.001, one-way ANOVA Tukey test.

**Supplementary Figure 4** Characterization of patient-derived iPSCs. (a) Immunofluorescence staining for pluripotency markers (NANOG, SOX2, SSEA4, OCT4, and TRA1–60) with Hoechst nuclear counterstaining in GD patient-derived iPSCs. (b) Alkaline phosphatase staining of iPSC colonies. (c) Sanger sequencing chromatogram confirming the GBA 1448T>C mutation (left) and GCase enzyme activity in control and GD iPSCs (right). Statistical significance was determined by *p<0.05, **p<0.01, ***p<0.001, one-way ANOVA Tukey test.

**Supplementary Figure 5** Plasma galectin-3 levels in patients with GD. (a) Age distribution of patients with GD (n = 7) and controls (n = 48) in whom plasma galectin-3 levels were measured. There was no significant difference in age between the two groups, as assessed by an unpaired *t*-test. (b) Plasma galectin-3 levels in patients with GD and controls. Plasma galectin-3 levels were significantly elevated in patients with GD (*p< 0.05).

**Supplementary Table S1.** Cell-type, cluster, and microglial state composition in the snRNA-seq dataset. Cell-type and cluster composition across conditions, including whole-cortex cluster abundance, microglial subcluster distribution, pseudotime summaries, and trajectory-state occupancy.

**Supplementary Table S2.** Differential expression and GD-associated gene analyses. Complete differential-expression results across major cortical cell types, cell type–specific disease perturbation scores, and expression statistics for the 21-gene DAM/microglial activation and senescence/SASP marker panel.

**Supplementary Table S3.** Pathway, module, and transcription factor activity analyses.

Gene sets and complete results for pathway module scores, transcription factor activity inference, MA-5 rescue scores, and pathway enrichment analyses.

**Supplementary Table S4.** Gene signatures and signature score statistics. Gene sets used to calculate DAM, microglial activation, IFN response, NDMG, and SASP signature scores, together with cell type–specific signature score statistics across Control, GD, and GD+MA-5 conditions.

**Supplementary Table S5.** Microglial trajectory and pseudotime analyses.

Results of microglial trajectory analysis, including pseudotime-associated candidate genes, pseudotime-resolved gene-expression patterns, and trajectory-related comparisons between GD and GD+MA-5 microglia.

**Supplementary Table S6.** Transcriptomic age analyses. Complete tAge results across brain cell types and microglial states, including clock-specific effect estimates, associations with DAM, IFN, and SASP programs, trajectory-state tAge, and gene-level contributions to mortality-associated tAge.

**Supplementary Table S7.** Primer sequences used for quantitative PCR and DNA sequencing.

Primer sequences used for quantification of cytosolic mitochondrial DNA (mtDNA), reverse transcription quantitative PCR (RT–qPCR), and PCR amplification for Sanger sequencing are listed. Cytosolic mtDNA abundance was assessed using primers for mitochondrial cytochrome c oxidase I (COX1) and normalized to nuclear 18S ribosomal RNA DNA. Relative mRNA expression was normalized to GAPDH. All primer sequences are shown in the 5′ –3′ direction.

**Supplementary movie 1** Video recordings of the rotarod test showing motor function in three experimental groups: Control (top), GD model (middle), and GD model treated with MA-5 (bottom). The control mouse demonstrates normal balance and motor coordination on the rotating rod. The GD model mouse exhibits impaired motor function and difficulty maintaining position on the rod. The MA-5-treated GD mouse shows improved motor function compared to the untreated GD model, supporting the findings described in Figure 1e regarding MA-5’s ability to improve neuromotor function in the GD model.

**Supplementary movie 2** Time-lapse fluorescence microscopy video showing mitochondrial dynamics in fibroblasts stained with MitoTracker Green. The three panels represent different experimental conditions: control, GD (middle), and GD treated with MA-5 (bottom). This video demonstrates the impaired mitochondrial mobility in GD fibroblasts and its restoration following MA-5 treatment, corresponding to findings discussed in Figure 6f.

Supplementary Files

This is a list of supplementary files associated with this preprint. Click to download.

- Svideo13GDMA5timelapse.mov
- Svideo13GDMA5timelapse.mov
- Svideo13GDMA5timelapse.mov
- Svideo13GDMA5timelapse.mov
- 4.SupplementaryFigure15v14.pdf
- 4.SupplementaryFigure15v14.pdf
- 4.SupplementaryFigure15v14.pdf
- 4.SupplementaryFigure15v14.pdf
- Svideo12GDtimelapse.mov
- Svideo12GDtimelapse.mov
- Svideo12GDtimelapse.mov
- Svideo12GDtimelapse.mov
- Svideo11Controltimelapse.mp4
- Svideo11Controltimelapse.mp4
- Svideo11Controltimelapse.mp4
- Svideo11Controltimelapse.mp4
- SupplementaryTable17v2.xlsx
- SupplementaryTable17v2.xlsx
- SupplementaryTable17v2.xlsx
- SupplementaryTable17v2.xlsx
- Svideo22GD.mov
- Svideo22GD.mov
- Svideo22GD.mov
- Svideo22GD.mov
- Svideo23GDMA5.mov
- Svideo23GDMA5.mov
- Svideo23GDMA5.mov
- Svideo23GDMA5.mov
- Svideo21control.mov
- Svideo21control.mov
- Svideo21control.mov
- Svideo21control.mov

## Figures and Tables

**Table 1:** Clinical and genetic characteristics of patients with Gaucher disease. The table summarizes Gaucher disease (GD) type, sex, GBA1 genotype, age at disease onset, and age at sample collection. Ages are expressed in years.

	Type	sex	gene mutation	age of onset	age
Patient 1	1	Male	L444P/L444P	1	23
Patient 2	2	Female	L444P(c.1448T>C/T)	10	10
Patient 3	2	Male	L444P(c.1448T>C)	18	18
Patient 4	2	Male	V230G/R296X	6	6

## Data Availability

RNA-seq data are deposited under accession numbers PRJNA1177609 and PRJNA1177610.

## Abbreviations:

8-OHdG: 8-hydroxy-2′-deoxyguanosine
ABR: auditory brainstem response test
BBB: blood–brain barrier
CBE: conduritol-B-epoxide
cGAS: cyclic GMP–AMP synthase
DAM: disease-associated microglia
ECAR: extracellular acidification rate
GBA: glucocerebrosidase
GCase: acid β-glucosidase
GD: Gaucher disease
GDF-15: growth differentiation factor 15
GlcCer: glucosylceramide
Glc-Sph: glucosylsphingosine
GSEA: gene set enrichment analysis
IFN: interferon
IL: interleukin
iPSC: induced pluripotent stem cell
ISG: interferon-stimulated gene
m1A: 1-methyladenosine
MA-5: Mitochonic Acid-5
mtDNA: mitochondrial DNA
mtROS: mitochondrial reactive oxygen species
NDMG: neurodegenerative microglia
NLRP3: NOD-like receptor family pyrin domain containing 3
OCR: oxygen consumption rate
OPC: oligodendrocyte precursor cell
OXPHOS: oxidative phosphorylation
PCA: principal component analysis
PD: Parkinson’s disease
pSTING: phosphorylated STING
ROS: reactive oxygen species
SASP: senescence-associated secretory phenotype
snRNA-seq: single-nucleus RNA sequencing
STING: stimulator of interferon genes
tAge: transcriptomic age
UMAP: Uniform Manifold Approximation and Projection
